# Effect of the Counterion on Circularly Polarized Luminescence of Europium(III) and Samarium(III) Complexes

**DOI:** 10.1021/acs.inorgchem.0c00280

**Published:** 2020-03-18

**Authors:** Lorenzo Arrico, Chiara De Rosa, Lorenzo Di Bari, Andrea Melchior, Fabio Piccinelli

**Affiliations:** † Dipartimento di Chimica e Chimica Industriale, Università di Pisa, via Moruzzi 13, 56124 Pisa, Italy; ‡ Luminescent Materials Laboratory, DB, Università di Verona, and INSTM, University of Verona Research Unit, Strada Le Grazie 15, 37134 Verona, Italy; § Laboratorio di Tecnologie Chimiche, Dipartimento Politecnico di Ingegneria e Architettura, Università di Udine, via Cotonificio 108, 33100 Udine, Italy

## Abstract

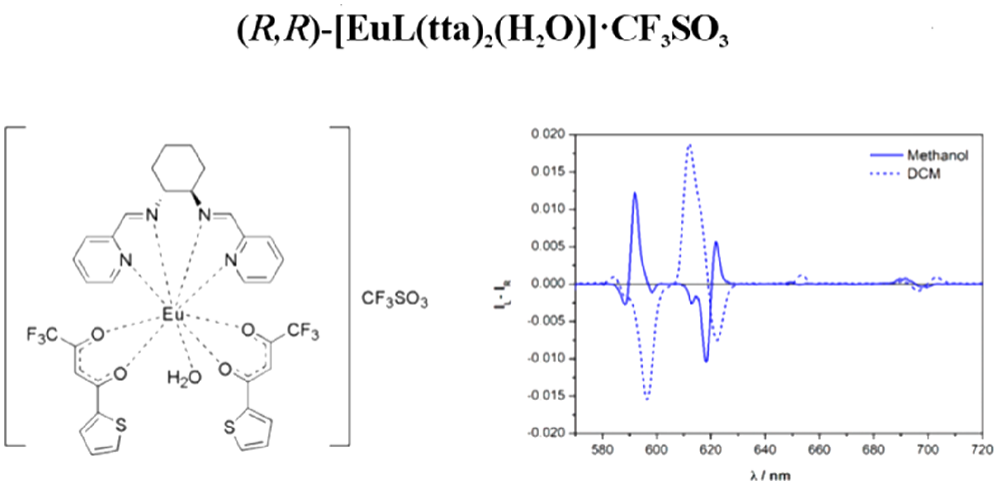

Each enantiopure europium(III) and samarium(III) nitrate and triflate complex of the ligand **L**, with **L** = *N*,*N*′-bis(2-pyridylmethylidene)-1,2-(*R*,*R* + *S*,*S*)-cyclohexanediamine ([Ln**L**(tta)_2_]·NO_3_ and [Ln**L**(tta)_2_(H_2_O)]·CF_3_SO_3_, where tta = 2-thenoyltrifluoroacetylacetonate) has been synthesized and characterized from a spectroscopic point of view, using a chiroptical technique such as electronic circular dichroism (ECD) and circularly polarized luminescence (CPL). In all cases, both ligands are capable of sensitizing the luminescence of both metal ions upon absorption of light around 280 and 350 nm. Despite small differences in the total luminescence (TL) and ECD spectra, the CPL activity of the complexes is strongly influenced by a concurrent effect of the solvent and counterion. This particularly applies to europium(III) complexes where the CPL spectra in acetonitrile can be described as a weighed linear combination of the CPL spectra in dichloromethane and methanol, which show nearly opposite signatures when their ligand stereochemistries are the same. This phenomenon could be related to the presence of equilibria interconverting solvated, anion-coordinated complexes and isomers differing by the relative orientation of the tta ligands. The difference between some bond lengths (M–N bonds, in particular) in the different species could be at the basis of such an unusual CPL activity.

## Abstract 2

Triflate ([Eu**L**(tta)_2_(H_2_O)]·CF_3_SO_3_) and nitrate ([Eu**L**(tta)_2_]·NO_3_) complexes, with **L** = *N*,*N*′-bis(2-pyridylmethylidene)-1,2-(*R*,*R* or *S*,*S*)-cyclohexanediamine, where tta = 2-thenoyltrifluoroacetylacetonate, show nearly opposite circularly polarized luminescence (CPL) signatures when dissolved in dichloromethane (DCM) or methanol (MeOH), even though their ligand stereochemistries remain unchanged. The presence (in DCM) and absence (in MeOH) of the counterion in the inner coordination sphere determine a strong change of the CPL activity of the relative europium(III) complex.

## Introduction

Circularly polarized luminescence (CPL) is a chiroptical phenomenon by which a luminescent compound or material emits different intensities of left and right circularly polarized light at a specific wavelength after excitation with unpolarized light.^1−6^ In order to define quantitatively the importance of this phenomenon, the luminescence dissymmetry factor *g*_lum_ is calculated, which is defined as follows: *g*_lum_ = 2(*I*_L_ – *I*_R_)/(*I*_L_ + *I*_R_), with *I*_L_ and *I*_R_ being the left and right polarized intensity, respectively. Circular polarization of the emitted light offers great potential for applications, such as in bioimaging^7^ and biosensing.^8−11^ Another field where CPL plays a pivotal role is that of organic light-emitting diodes.^12−14^ For similar applications, sizable values of *g*_lum_ are required. In this context, lanthanide ion emission in lanthanide-based complexes may reach high *g*_lum_ values (between 0.1 and 1.45),^1−6,15−18^ and this is due to the intrinsic nature of their f–f transitions, which are magnetic-dipole-allowed and electric-dipole-forbidden. Following Richardson’s classification,^19^ sizable values of *g*_lum_ are expected for europium(III) and terbium(III) in particular, even though samarium(III) and dysprosium(III) should also be considered. Because of the fact that samarium(III) is more sensitive to the multiphonon relaxation process, its complexes are only weakly luminescent, and for this reason, they have been poorly studied in the past. To the best of our knowledge, to date, only a few samarium complexes were described to exhibit CPL in solution.^13,20−26^ In all cases, in order to mitigate the multiphonon relaxation process negatively affecting the luminescence emission efficiency, the donor atoms should not bear any H atoms.

**Figure 1:**
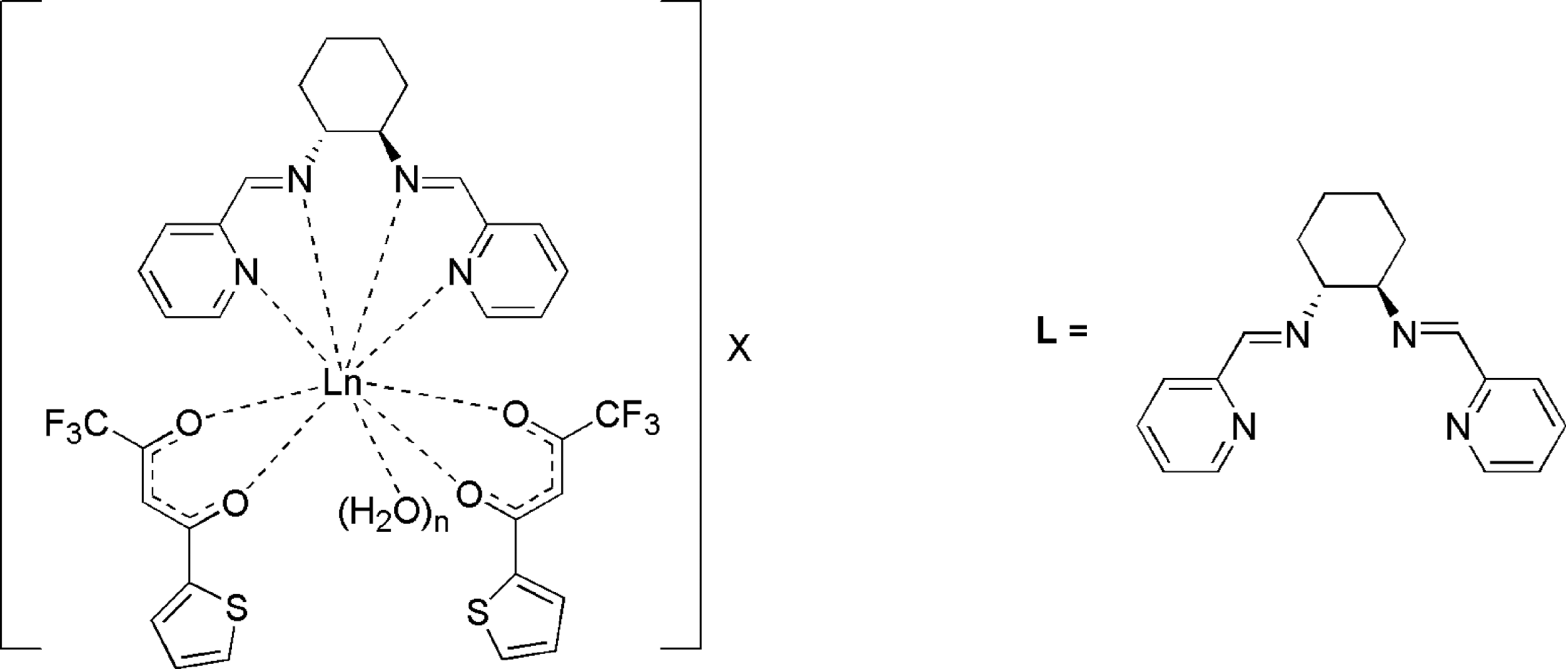
Molecular structure of the complexes under investigation in the present contribution. Ln = Sm and Eu; X = NO_3_ and CF_3_SO_3_; *n* = 0 or 1. Both enantiomers of the ligand have been employed.

Recently, a paper by Wada et al.^27^ attracted our attention. They demonstrated that the chiral geometric environment around europium(III) and also its CPL signature can undergo substantial changes depending on the addition of further achiral molecules (acetone or triphenylphosphine oxide), which coordinate the metal ion. This clearly demonstrated that the contributions of both chiral and achiral ligands must be considered, where chiroptical activity such as CPL is concerned. In this direction, some of us^28^ discovered the interesting role of another achiral entity [the solvent: acetonitrile (AN) vs methanol (MeOH)] in the definition of the final CPL signature of a chiral europium(III) complex. In order to gain more insight into the exact role (direct or indirect) of the solvent in influencing the CPL signal, we synthesized similar europium(III) complexes with different counterions (triflate or nitrate; Figure 1 and Table 1) and measured their CPL spectra in different solvents [i.e., AN, MeOH, and dichloromethane (DCM)]. A similar study has been performed on analogous samarium(III) complexes (Figure 1 and Table 1) and also for the purpose of enlarging the repertory of samarium complexes exhibiting CPL.

**Table 1:** Labels of the Complexes Discussed in This Paper^a^

	X	
Ln	NO_3_ (nitrate)	CF_3_SO_3_ (triflate)
Eu	[Eu**L**(tta)_2_]·NO_3_	[Eu**L**(tta)_2_(H_2_O)]·CF_3_SO_3_
Sm	[Sm**L**(tta)_2_]·NO_3_	[Sm**L**(tta)_2_(H_2_O)]·CF_3_SO_3_

a tta = 2-thenoyltrifluoroacetylacetonate.

## Experimental Section

Eu(CF_3_SO_3_)_3_, Sm(CF_3_SO_3_)_3_, Eu(NO_3_)_3_·6H_2_O, and Sm(NO_3_)_3_·6H_2_O (Aldrich, 98%) were stored under vacuum for several days at 80 °C and then transferred in a glovebox.

*N*,*N*′-Bis(2-pyridylmethylidene)-1,2-(*R*,*R* + *S*,*S*)-cyclohexanediamine (**L**; Figure 1) were synthesized by following the procedures reported in the literature.^29,30^ [EuL(tta)_2_(H_2_O)]·CF_3_SO_3_ was synthesized as reported in the literature.^28^

[Eu**L**(tta)_2_]·NO_3_ was synthesized as follows: at room temperature, 76 mg (0.342 mmol) of 2-thenoyltrifluoroacetylacetone (Htta) was dissolved in a MeOH (1.5 mL) solution containing 19 mg (0.342 mmol) of KOH. The clear solution was slowly added to a MeOH solution (2 mL) of the enantiopure ligand **L** [50 mg (0.171 mmol)] and Eu(NO_3_)_3_·6H_2_O [76.4 mg (0.171 mmol)]. The final mixture was stirred for 30 min at room temperature, and then the solvent was removed under reduced pressure. The desired product was obtained in good yield as a yellowish powder upon extraction in DCM (6 mL), followed by solvent removal under reduced pressure. [Eu**L**(tta)_2_]·NO_3_: yield in the 88–92% range for the two enantiomers. Elem anal. Calcd for C_34_H_28_EuF_6_N_5_O_7_S_2_ (MW = 948.7): C, 43.04; H, 2.97; N, 7.38; O, 11.81. Found: C, 42.87 ; H, 2.90; N, 7.26; O, 11.87 (isomer *R*,*R*); C, 42.81 ; H, 2.88; N, 7.28; O, 11.96 (isomer *S*,*S*). In AN: ε (279 nm) = 27290 and 27003 M^–1^ cm^–1^ (pyridine ring absorption) for *R*,*R* and *S*,*S* isomers, respectively; ε (347 nm) = 35570 and 35300 M^–1^ cm^–1^ (tta absorption) for *R*,*R* and *S*,*S* isomers, respectively.

[Sm**L**(tta)_2_(H_2_O)]·CF_3_SO_3_ was synthesized as follows: at room temperature, 53.3 mg (0.240 mmol) of Htta were dissolved in a MeOH (1.5 mL) solution containing 13.5 mg (0.240 mmol) of KOH. The clear solution was slowly added to a MeOH solution (1.5 mL) of the ligand **L** [35 mg (0.120 mmol)] and Sm(CF_3_SO_3_)_3_ [71.6 mg (0.120 mmol)]. The final mixture was stirred for 1 h at room temperature, and then the solvent was removed under reduced pressure. The desired product was obtained in good yield as a yellowish powder upon extraction in DCM (5 mL), followed by removal of the solvent under reduced pressure. The synthesis was performed by using both enantiomers of the ligand **L**. [Sm**L**(tta)_2_(H_2_O)]·CF_3_SO_3_: yield 84%. Elem anal. Calcd for C_35_H_30_F_9_N_4_O_8_S_3_Sm (MW = 1052.2): C, 39.95; H, 2.87; N, 5.32; O, 12.16. Found: C, 39.80 ; H, 2.98; N, 5.25; O, 12.09 (isomer *R*,*R*); C, 39.78 ; H, 2.86; N, 5.37; O, 11.96 (isomer *S*,*S*). In AN: ε (280 nm) = 26560 and 26980 M^–1^ cm^–1^ (pyridine ring absorption) for *R*,*R* and *S*,*S* isomers, respectively; ε (347 nm) = 34877 and 35112 M^–1^ cm^–1^ (tta absorption) for *R*,*R* and *S*,*S* isomers, respectively.

[Sm**L**(tta)_2_]·NO_3_ was synthesized as follows: at room temperature, 53.3 mg (0.240 mmol) of Htta was dissolved in a MeOH (1.5 mL) solution containing 13.5 mg (0.240 mmol) of KOH. The clear solution was slowly added to a MeOH solution (1.5 mL) of the ligand **L** [35 mg (0.120 mmol)] and Sm(NO_3_)_3_·6H_2_O [53.3 mg (0.120 mmol)]. The final mixture was stirred for 1 h at room temperature, and then the solvent was removed under reduced pressure. The desired product was obtained in good yield as a yellowish powder upon extraction in DCM (5 mL), followed by removal of the solvent under reduced pressure. [Sm**L**(tta)_2_]·NO_3_: yield 95%. Elem anal. Calcd for C_34_H_28_F_6_N_5_O_7_S_2_Sm (MW = 947.1): C, 43.12; H, 2.98; N, 7.39; O, 11.83. Found: C, 42.94 ; H, 2.90; N, 7.33; O, 11.69 (isomer *R*,*R*); C, 42.99 ; H, 2.79; N, 7.21; O, 11.80 (isomer *S*,*S*). In AN: ε (279 nm) = 26750 and 27010 M^–1^ cm^–1^ (pyridine ring absorption) for *R*,*R* and *S*,*S* isomers, respectively; ε (347 nm) = 34870 and 35320 M^–1^ cm^–1^ (tta absorption) for *R*,*R* and *S*,*S* isomers, respectively.

### Luminescence and Decay Kinetics

Room temperature luminescence was measured with a Fluorolog 3 (Horiba-Jobin Yvon) spectrofluorometer, equipped with a xenon lamp, a double excitation monochromator, a single emission monochromator (model HR320), and a photomultiplier in photon counting mode for detection of the emitted signal. All of the spectra were corrected for spectral distortions of the setup. The spectra were recorded on AN (0.4 mM) and MeOH (0.4 mM) solutions, as for the CPL spectra (see below).

In decay kinetics measurements, a xenon microsecond flashlamp was used, and the signal was recorded by means of a multichannel scaling method. True decay times were obtained using convolution of the instrumental response function with an exponential function and a least-squares-sum-based fitting program (*SpectraSolve* software package).

### CPL

CPL spectra were recorded with the homemade spectrofluoropolarimeter described previously.^31^ The spectra were recorded on AN (0.4 mM), MeOH (0.4 mM), and DCM (0.4 mM) solutions in a 1 cm cell. The samples were excited at 365 nm, with a 90° geometry between the detector and light source.

### Electronic Circular Dichroism (ECD)

ECD spectra were recorded with a Jasco J710 spectropolarimeter on 2 mM AN and 2 mM MeOH solutions in a 0.02 cm cell.

### NMR

^1^H NMR spectra were acquired on a Bruker DRX 400 spectrometer, using the residual solvent peaks as internal references.

### Density Functional Theory (DFT) Calculations

Because the paramagnetic europium(III) and samarium(III) complexes are difficult to model computationally, the diamagnetic and lighter yttrium(III) analogues were studied. It has been shown that yttrium(III) complexes may serve as suitable models for the europium(III) analogues.^32^ Geometry optimizations of the [Y**L**(tta)_2_]·X (where X = NO_3_ or CF_3_SO_3_ anions) complexes were carried out at the DFT level in a vacuum using the B3LYP^33,34^ exchange–correlation functional. The 6-31+G(d) basis set was employed for the ligand atoms, while Y^III^ ion was described by the quasi-relativistic small-core Stuttgart–Dresden pseudopotential and relative basis set.^35^ All final structures were checked as minima by vibrational analysis. Geometry optimizations were repeated including solvent effects by means of the polarizable continuum model method^36^ in DCM and MeOH. The configurational isomers of the complexes depicted in Figure S1 were considered to check the presence of isomerization equilibria associated with different relative orientations of the tta ligands.

Isomer A was found in the crystal structure.^28^ To reduce the computational cost, the F atoms of tta were replaced with H atoms. The free energies for the solvent ligand-exchange reactions in MeOH were calculated by applying corrections for the standard-state change from the gas to solution phase for the reagents and products.^37^ All calculations were carried out with *Gaussian16*.^38^

### Elemental Analysis

Elemental analyses were carried out by using a EACE 1110 CHNO analyzer.

## Results and Discussion

### UV–Visible Absorption and ECD

**Figure 2:**
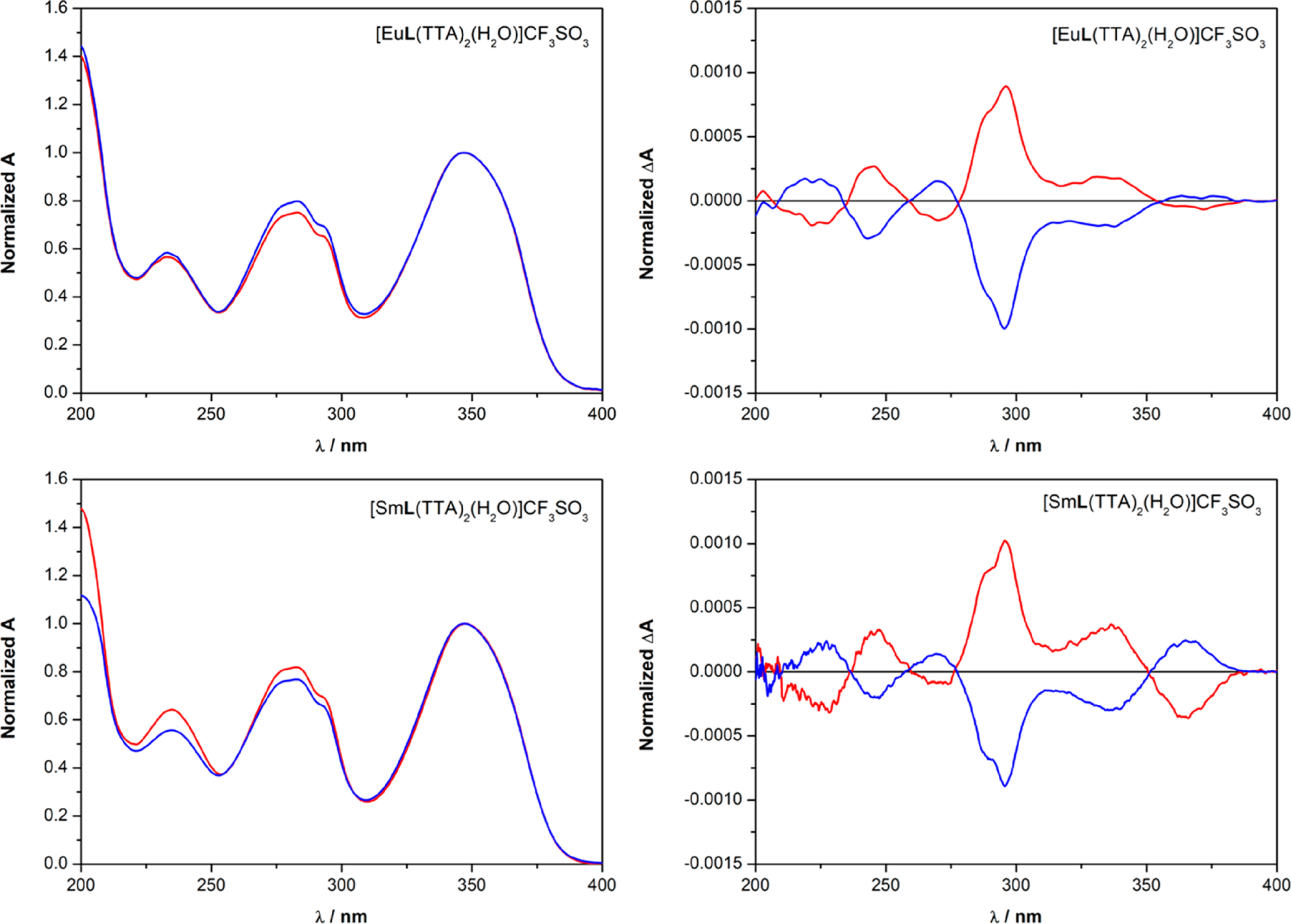
UV–visible absorption (left) and ECD (right) spectra of [Eu**L**(tta)_2_(H_2_O)]·CF_3_SO_3_ (top) and [Sm**L**(tta)_2_(H_2_O)]·CF_3_SO_3_ (bottom) in AN. The spectra of the *R*,*R* enantiomers are reported in blue, while the spectra of the *S*,*S* enantiomers are reported in red. Both UV–visible and ECD spectra are normalized on the maximum absorbance value of the band centered at 350 nm.

The UV–visible electronic absorption and ECD spectra of the triflate complexes ([Eu**L**(tta)_2_(H_2_O)]CF_3_SO_3_ and [Sm**L**(tta)_2_(H_2_O)]CF_3_SO_3_) in AN are reported in Figure 2, and their features are, in practice, independent of the employed metal ion (Sm or Eu).

This finding is in agreement with the ligand-centered nature of the involved electronic transitions. In fact, the strongest peaks at 285 and 350 nm are assigned to the overlapping absorption bands of the **L** and tta ligands bound to the metal ion, respectively. As was already discussed, the absorption band around 350 nm can be attributed to the diketonate-centered singlet–singlet π–π* enolic transition,^39^ while the composite absorption band peaking around 280 nm is assigned to the electronic transitions involving both the pyridine ring and conjugated C=N group (i.e., π–π* and n−π* transitions) of the ligand **L**.^28^ The sign of the ECD bands reflects the stereochemistry of the chiral ligand **L**, which is also capable of dictating a preferred sense of twist of the diketonates, as demonstrated by a dichroic signal around 350 nm, where the absorption of tta takes place. The dichroic band around 370 nm would suggest a positive coupling for the *R*,*R* enantiomers for both europium and samarium, i.e., a positive (clockwise) arrangement of the diketonates. Small differences in both the absorption and ECD spectra are detected between samarium and europium, upon changing the solvent from AN to MeOH, by using nitrate instead of triflate as a counteranion (Figures S2 and S3). These slight discrepancies suggest some minor structural rearrangements due to the different lanthanide ion, solvent, and counterion.

### Total Luminescence (TL), CPL, ^1^H NMR, and Luminescence Decay Kinetics

#### Europium Complexes

**Figure 3:**
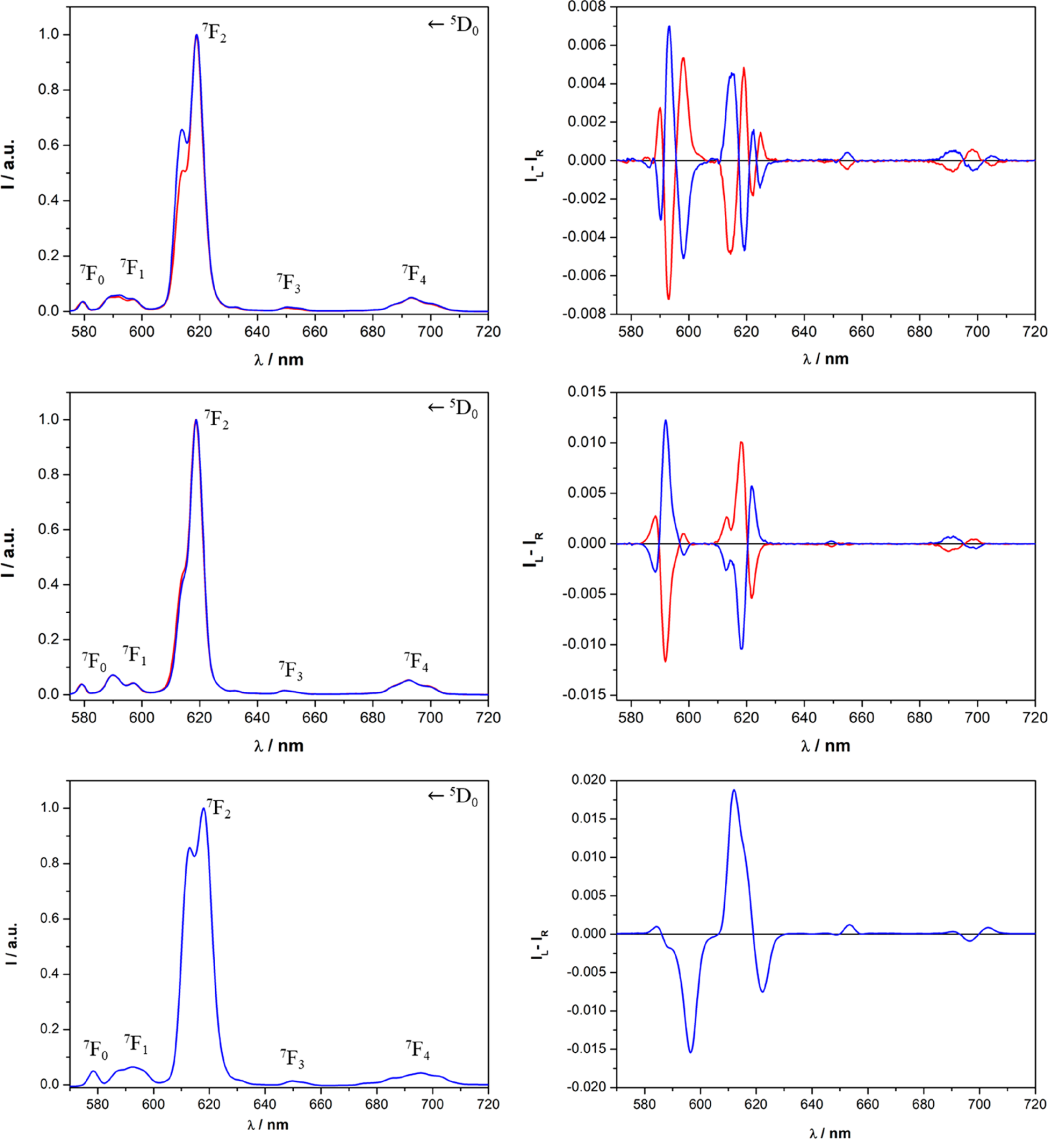
TL (left) and CPL (right) spectra of the [Eu**L**(tta)_2_(H_2_O)]·CF_3_SO_3_ complex dissolved in AN (top), MeOH (middle), and DCM (bottom) (λ_exc_ = 365 nm). The spectra of the *R*,*R* enantiomer are reported in blue, while the spectra of the *S*,*S* enantiomer are reported in red. Both the TL and CPL intensities are normalized on the maximum of the ^5^D_0_ → ^7^F_2_ transition. For a clear visual comparison of the CPL spectra upon changes of the solvent, in the case of the *S*,*S* enantiomer dissolved in DCM, the spectrum is omitted. However, it is the perfect mirror image of the spectrum recorded for the *R*,*R* isomer in this same solvent.

**Figure 4:**
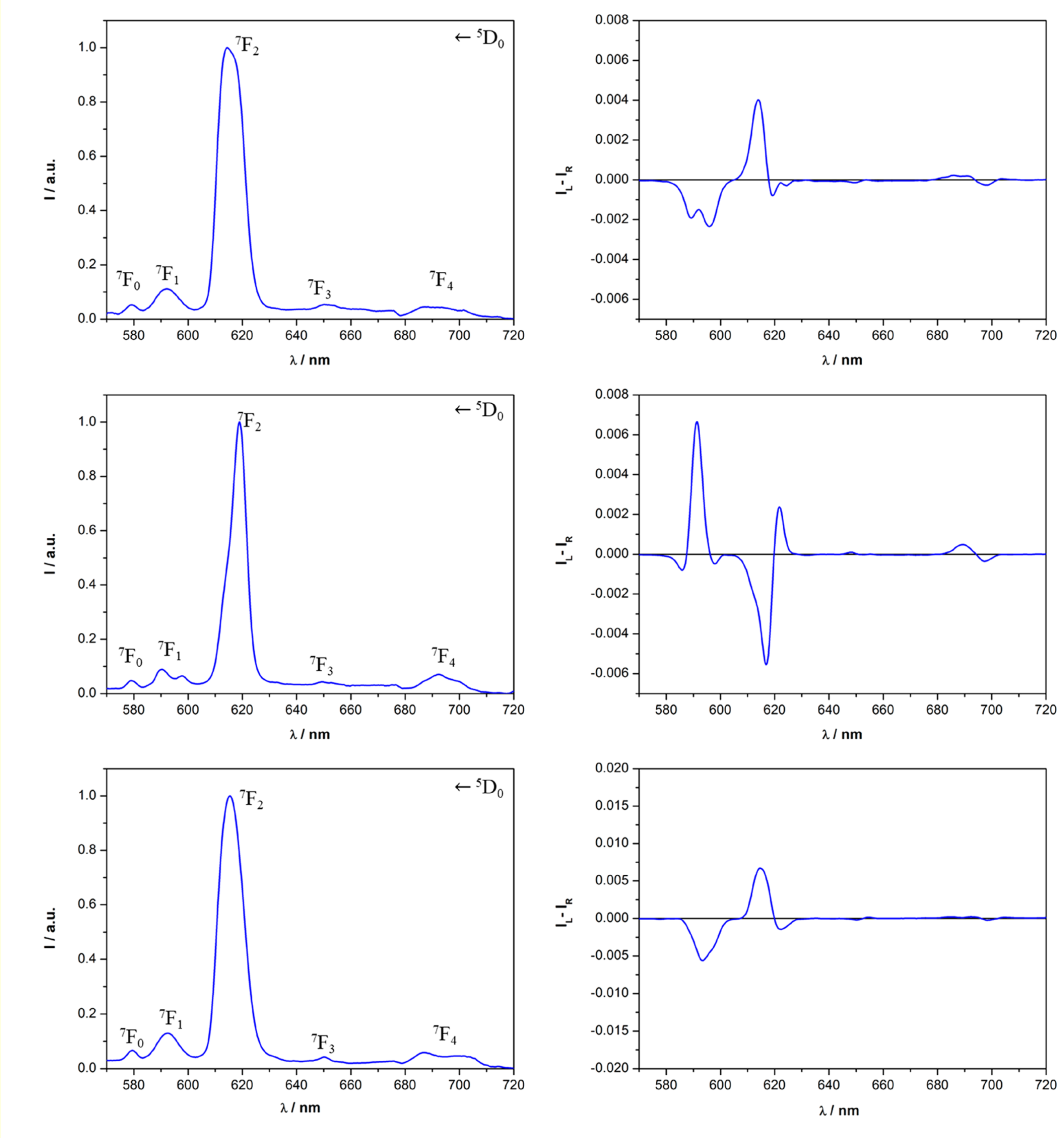
TL (left) and CPL (right) spectra of the [Eu**L**(tta)_2_]·NO_3_ complex dissolved in AN (top), MeOH (middle), and DCM (bottom) (λ_exc_ = 365 nm). Both the TL and CPL intensities are normalized on the maximum of the ^5^D_0_ → ^7^F_2_ transition. The ligand **L** has *R*,*R* stereochemistry. For a clear visual comparison of the CPL spectra in different solvents, those for the *S*,*S* enantiomer are omitted. In all cases, they are the perfect mirror images of the CPL spectra recorded for the *R*,*R* isomer.

The europium(III) TL spectra of the triflate and nitrate complexes ([Eu**L**(tta)_2_(H_2_O)]·CF_3_SO_3_ and [Eu**L**(tta)_2_]·NO_3_) are compatible with an emitting Eu^III^ ion surrounded by a crystal field whose geometry deviates significantly from the inversion symmetry because the ^5^D_0_ → ^7^F_2_ transition dominates the spectra (Figures 3 and 4). This is compatible with the overall chirality of the complex, discussed in the previous section. For both anions, the typical red luminescence of europium(III) is effectively sensitized upon excitation of both the **L** (peak around 280 nm) and tta (peak around 350 nm) ligands. Although the TL spectra of the complexes display only minor differences upon changes of the solvent and counterion, we noticed strong differences in the CPL spectra. As shown in Figure 3, the CPL signatures of the two enantiomers of the triflate complexes are perfect mirror images in all of the employed solvents, but they are strongly dependent on the solvent.

It is particularly striking that [Eu**L**(tta)_2_(H_2_O)]·CF_3_SO_3_ possessing the *same ligand stereochemistry* shows CPL spectra that are nearly inverted when it is dissolved in MeOH and DCM (Figures 3 and S4). Moreover, for the ^5^D_0_ →^7^F_2_ transition, we observed three bands in MeOH and four bands, two positive and two negative, in AN. As far as the nitrate complex [Eu**L**(tta)_2_]·NO_3_ in which the ligand **L** possesses *R*,*R* stereochemistry is concerned, we observed the same behavior as that described for the triflate complex in MeOH and DCM (Figure 4). In contrast to the CPL spectrum of [Eu**L**(tta)_2_(H_2_O)]·CF_3_SO_3_ in AN, that of [Eu**L**(tta)_2_]·NO_3_ in this same solvent is more similar to the spectrum of this nitrate complex recorded in DCM (Figure 4).

**Figure 5:**
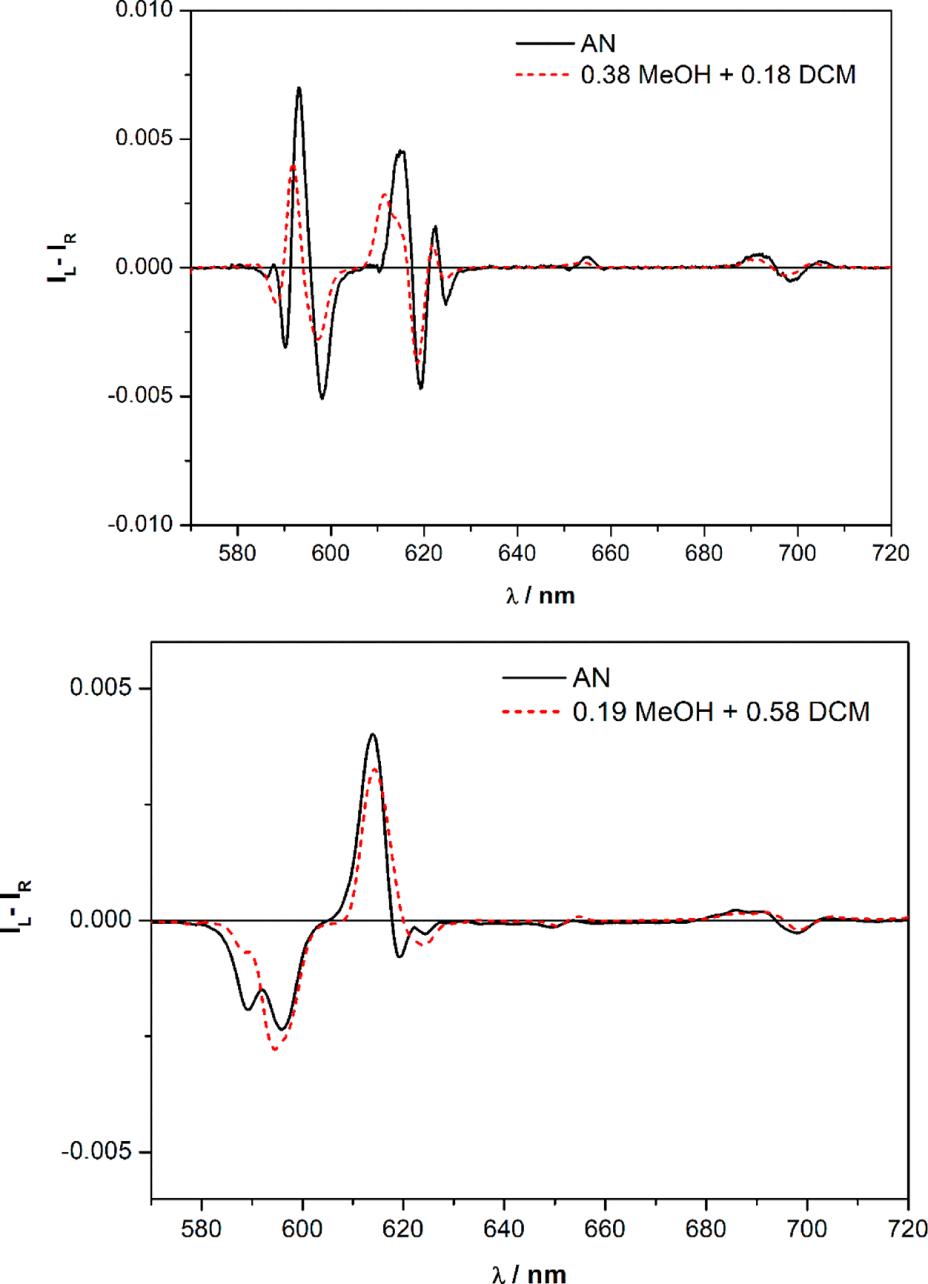
Top: CPL spectra of (*R*,*R*)-[Eu**L**(tta)_2_(H_2_O)]·CF_3_SO_3_ when dissolved in AN (black line) and as a linear combination of the CPL signature in MeOH and DCM (red dashed line). Bottom: CPL spectra of (*R*,*R*)- [Eu**L**(tta)_2_]·NO_3_ when dissolved in AN (black line) and as a linear combination of the CPL signature in MeOH and DCM (red dashed line).

It is evident that the nature of both the solvent and counterion plays a crucial role in the determination of the CPL activity of the complex. Interestingly, in the case of both the europium triflate and nitrate complexes, the CPL spectra of one enantiomer in AN is almost superimposable on the weighed linear combinations of two CPL spectra of the same complex (and same enantiomer) recorded in MeOH and DCM (Figure 5). This observation suggests that the complex in AN (mean polarity solvent) would be depicted as a weighted combination of the situation in MeOH (very polar) and DCM (apolar).

At least in the case of [Eu**L**(tta)_2_(H_2_O)]·CF_3_SO_3_, which displays very distinct ^1^H NMR signals, this finding is strongly supported by analysis of the chemical shifts recorded in the three solvents. In particular, the experimental ^1^H NMR chemical shifts in AN-*d*_3_ retrace the chemical shifts calculated as a linear combination of the experimental chemical shifts of the complex recorded in MeOH-*d*_4_ and DCM-*d*_2_, using *the same molar fractions* as those obtained through analysis of the CPL spectra, as shown in Table S1. In the case of [Eu**L**(tta)_2_]·NO_3_, the ^1^H NMR spectra are more complex (Figures S9–S11); therefore, we were not able to perform the same analysis.

We assume that, in apolar noncoordinating solvents such as DCM, both triflate and nitrate anions are directly bound to the metal cation. This evidence is supported by the luminescence decay study discussed later and is in agreement with the literature, where examples about competition in the coordination to the metal center between these anions and DCM are not reported. This is not the case of the polar and protic MeOH, which has been proposed to have a solvation strength intermediate between AN and dimethylformamide.^40^ In AN, it is known that nitrate salts of Ln^3+^ ions act as *non*electrolytes,^40^ and also in aqueous MeOH, it is has been shown that weak complexes are formed.^41^ The triflate anion is known to form complexes with Ln^3+^ ions in both anhydrous MeOH and AN^42,43^ even though the lanthanide triflates are often considered good electrolytes in AN. However, triflates have been shown to be completely dissociated in anhydrous AN at concentrations lower than 0.05 mM.^44^ Also, for complexes with **L**, it has been shown that the solvent nature deeply affects the nature of the adducts formed with a variety of ions.^45^ In light of these results, it is reasonable to connect the two different CPL signatures (one almost the mirror image of the other; Figures 3 and 4) with the degree of dissociation of the anions in the different solvents. In AN, the CPL spectral analysis suggests that (1) in this solvent coexist the species present in both MeOH and DCM and (2) their relative amounts depend on the anion. In particular, in the case of triflate, there is a prevailing presence of the dominant species present in MeOH (dissociated), while in the case of nitrate, the situation is the opposite (a qualitative comparison can be made by looking at the different coefficients of the two linear combinations in Figure 5).

It is remarkable that such profound changes in the metal-centered chiroptical property, namely, CPL, are not paralleled in the ligand-centered ECD, where all of the spectra are closely similar. We must recall that presently the ECD spectrum is dominated by the exciton coupling between ligands bonded to the same Eu^III^ ion, and the contribution due to the intrinsic chirality of **L** is negligibly small. The exciton coupling mechanism is very sensitive to the mutual orientation of the chromophoric ligands,^46^ and altogether this means that the overall structure of the complex is rather independent of the solvent or anion. Thus, the organic part of the coordination sphere must remain substantially the same, while the crystal field of lanthanide(III) is deeply affected by the bonded/nonbonded anion. In other terms, the large variation in CPL is a consequence of modulation of the various *M*_*J*_ components of each spectroscopic term, in terms of energy and possibly also in terms of transition probability (i.e., electric and magnetic transition dipole moments).^47^ This modulation of the crystal-field parameters as a function of the ligand polarizability and charge is reminiscent of what has been observed for ytterbium(III) near-IR circular dichroism.^48^

As far as the degree of polarization of the emitted light and the decay kinetics of the ^5^D_0_ Eu^III^ excited state are concerned, the highest values of the luminescence dissymmetry factor *g*_lum_ for all of the europium(III) complexes are reported in Table 2, together with the observed excited-state lifetimes.

**Table 2:** Values of the Emission Dissymmetry Factor *g*_lum_ and ^5^D_0_ Eu^III^ Excited-State Lifetimes of the Europium(III) Complexes under Investigation Dissolved in Different Solvents^a^

	solvent					
	*g*_lum_			observed lifetime (ms)		
complex	DCM	MeOH	AN	DCM	MeOH/CD_3_OD	AN
(*R*,*R*)-[Eu**L**(tta)_2_(H_2_O)]·CF_3_SO_3_	–0.23	+0.17	+0.11	0.54(1)	0.57(1)/0.75(1)	0.44(1)
(*R*,*R*)-[Eu**L**(tta)_2_]·NO_3_	–0.05	+0.07	–0.02	0.53(1)	0.42(1)/0.52(1)	0.53(1)

a The *g*_lum_ values refer to the most intense component of the ^5^D_0_ →^7^F_1_ transition.

The europium(III) complex presenting triflate as the counterion shows higher luminescence dissymmetry factors with respect to the nitrate analogues. In more detail, the highest |*g*_lum_| is recorded for [Eu**L**(tta)_2_(H_2_O)]·CF_3_SO_3_ in DCM. Interestingly, the magnitude and signs of the *g*_lum_ factors retrace at a glance the chemical behavior of both the triflate and nitrate complexes in the three different solvents. In fact, the *R*,*R* enantiomers of the complexes present the highest negative *g*_lum_ values in DCM and the highest positive *g*_lum_ values in MeOH, while in AN, the *g*_lum_ factors reach an intermediate value. In particular, in the case of the triflate complex, *g*_lum_ is positive and closer to that recorded in MeOH, as expected given the low coordinating ability of the anion. On the other hand, *g*_lum_ for the nitrate complex in AN is negative and closer to the one recorded in DCM, thus indicating that the anion is essentially coordinated to the Ln ion.

All of the decay curves are well fitted by a single-exponential function (for nitrate complexes, see Figure S5; for triflate complexes, see ref (28)), and the lifetimes in MeOH and DCM, which represent the two extreme cases, are rather similar in the case of triflate complexes. As discussed in that work,^28^ the presence of one water molecule in the inner coordination sphere of the metal ion when the triflate complex is dissolved in AN is capable of reducing, by means of the multiphonon relaxation phenomenon, the value of the observed lifetime. In the case of nitrate complexes, it is interesting to note that the observed lifetimes in DCM and AN are almost equal (0.53 ms, Table 2). This finding is in agreement with the conclusions drawn by CPL spectroscopy: the same prevailing species, characterized by nitrate bound to the metal ion, should be present in these two solvents. The chelation of nitrate contributes to hindering access to the Eu^III^ ion by solvent molecules, and, consequently, the solvent (DCM or AN) does not show any influence on the lifetime value. Furthermore, the addition of 1 drop of D_2_O in the AN solution of the nitrate complex should increase the value of the europium(III) lifetime if water molecules are bound to the metal ion because, as a consequence of D_2_O/H_2_O exchange, high-energy vibrations (OH) capable of reducing the value of europium(III) lifetime observed by the multiphonon relaxation process are removed from the inner coordination sphere. Because, upon D_2_O addition, the lifetime values do not change significantly [0.50(1) vs 0.53(1) ms], the presence of bound water can be ruled out. When the [Eu**L**(tta)_2_]·NO_3_ complex is dissolved in deuterated MeOH (CD_3_OD), we detect an increase of the europium(III) lifetime value. From the equation reported in the literature,^49^ the number of bound MeOH molecules (*m*) can be obtained by *m* = 2.1(1/τ_obs,MeOH_ – 1/τ_obs,CD_3_OD_). As for the europium(III) triflate complex,^28^ the calculated value of *m* = 1.0(5) is compatible with the presence of one MeOH molecule in the inner coordination sphere of europium(III) and also for the nitrate compound dissolved in MeOH. Finally, the quite similar luminescence decay times, recorded for triflate and nitrate complexes dissolved in different solvents, are indicative of a similar intrinsic quantum yield (in the 50–70% range), already determined for [Eu**L**(tta)_2_(H_2_O)]·CF_3_SO_3_.^28^

#### Samarium Complexes

**Figure 6:**
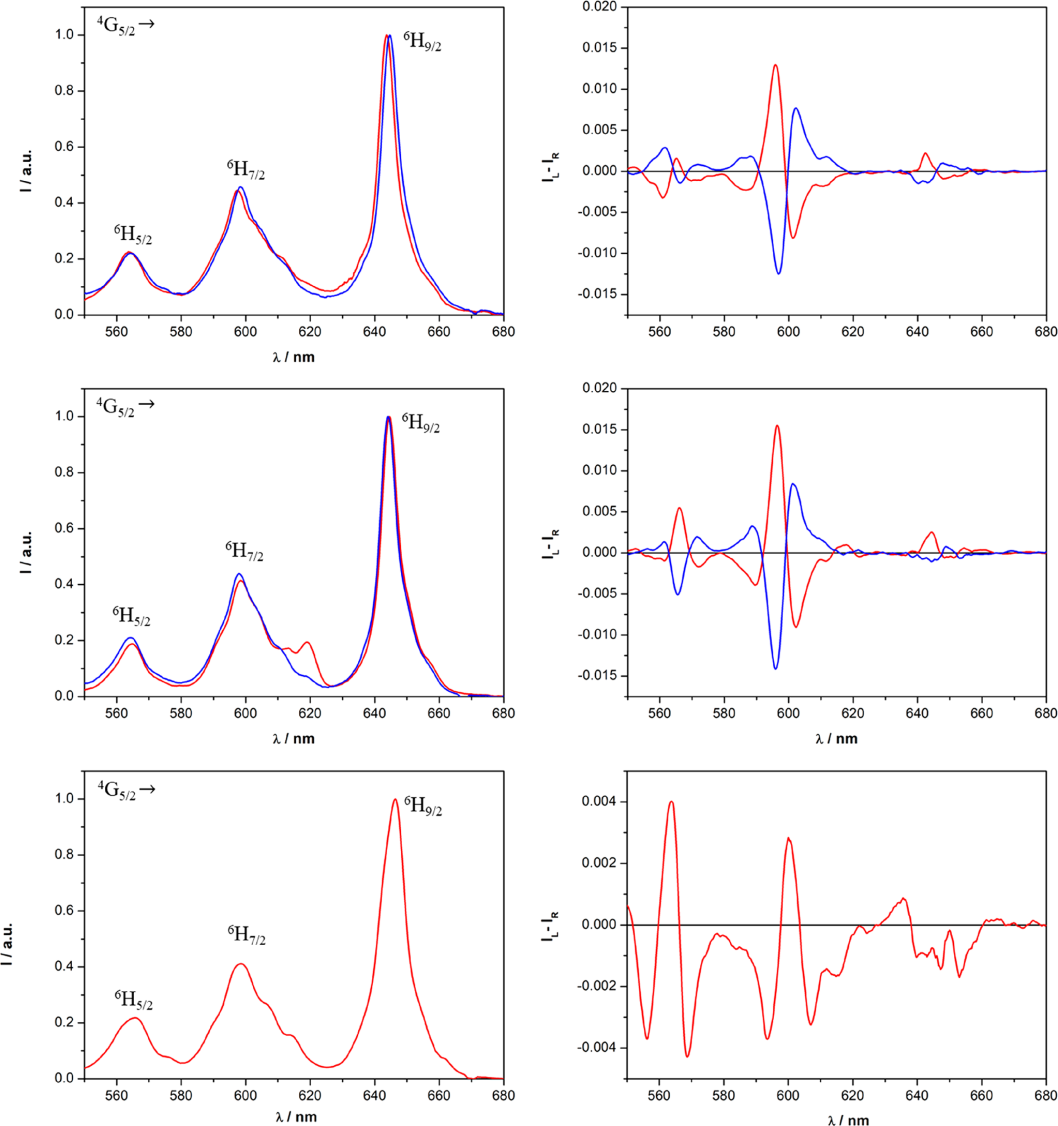
TL (leftt) and CPL (right) spectra of the [Sm**L**(tta)_2_(H_2_O)]·CF_3_SO_3_ complex dissolved in AN (top), MeOH (middle), and DCM (bottom) (λ_exc_ = 365 nm). The spectra of the *R*,*R* enantiomer are reported in blue, while the spectra of the *S*,*S* enantiomer are reported in red. Both the TL and CPL intensities are normalized on the maximum of the ^4^G_5/2_ → ^6^H_9/2_ transition. For a clear visual comparison of the CPL spectra upon changes in the solvent, in the case of *R*,*R* enantiomer dissolved in DCM, the spectrum is omitted. However, perfect mirror images of the spectra are recorded for the *S*,*S* isomer in this same solvent.

From inspection of the TL and CPL spectra (Figures 6 and S12), we can conclude that all samarium(III)-based complexes efficiently emit polarized light, in particular around 600 nm (corresponding to the ^4^G_5/2_ → ^6^H_7/2_ transition). Other than europium(III), the tta ligand (λ_exc_ = 365 nm) is capable of effectively transfering its excitation energy also to samarium(III).

In contrast to the analogous europium(III) complexes, independent of the solvent, the sequences of the signals in the CPL spectra of samarium(III) triflate complexes are quite similar. However, in AN and MeOH, the intensities of the CPL bands associated with the ^4^G_5/2_ → ^6^H_7/2_ (∼600 nm) transition are higher than those recorded in DCM. In the case of the CPL spectra of samarium(III) nitrate complexes (Figure S12), the main differences can be seen in the region centered around 560 nm (^4^G_5/2_ → ^6^H_5/2_): in AC and DCM, only one CPL band is present, while in MeOH, there are three CPL bands. These aspects can be related once again to the role of the counterion. Triflate and nitrate should be significantly coordinated to samarium(III) in DCM, while they should be preferentially dissociated in MeOH. In AN, however, the triflate ion is preferentially dissociated, while the nitrate ion is still preferentially coordinated to the metal center. The values of the luminescence dissymmetry factor *g*_lum_ and the observed excited-state lifetimes are reported in Table 3 (see also Figure S13).

**Table 3:** Values of the Emission Dissymmetry Factor *g*_lum_ and ^4^G_5/2_ Sm^III^ Excited-State Lifetimes of the Samarium(III) Complexes under Investigation Dissolved in Different Solvents^a^

	solvent					
	*g*_lum_			observed lifetime (μs)		
complex	DCM	MeOH	AN	DCM	MeOH/CD_3_OD	AN
(*S*,*S*)-[Sm**L**(tta)_2_(H_2_O)]·CF_3_SO_3_	+0.007	+0.035	+0.03	28.1(1)	17.9(1)/37.8(1)	25.2(1)
(*R*,*R*)-[Sm**L**(tta)_2_]·NO_3_	–0.016	–0.034	–0.015	28.3(1)	18.6(1)/32.6(1)	25.6(1)

a The *g*_lum_ values refer to the positive band of the ^4^G_5/2_ → ^6^H_7/2_ transition in the case of (*S*,*S*)-[Sm**L**(tta)_2_(H_2_O)]·CF_3_SO_3_ and to the negative band in the case of (*R*,*R*)-[Sm**L**(tta)_2_]·NO_3_.

The highest absolute value of *g*_lum_ is obtained for the complexes when they are dissolved in MeOH. In convest to the europium(III) complexes, in the case of samarium(III), with the sequence of the signals of the ^4^G_5/2_ → ^6^H_7/2_ transition being essentially the same, the signs of *g*_lum_ for the same enantiomer do not change in the three investigated solvents. As expected, the values of the |*g*_lum_| factors recorded in AN lie close to those recorded in MeOH in the case of the triflate complex and close to those recorded in DCM for the nitrate complex.

Also, the decay curves of the samarium(III) luminescence are well fitted by a single-exponential function. Because the values of the observed lifetimes fall in the microsecond range in all of the solvents, we can conclude that the samarium(III) emission efficiency is not so low even in nondeuterated solvents. In this context, it is useful to remind one that a good samarium(III) cryptate emitter shows a lifetime of around 90 μs in deuterated MeOH.^20^ Clearly, the **L** and tta ligands can effectively protect the metal ion from the intrusion of solvent molecules capable of activating the multiphonon relaxation mechanism. Unlike the analogue europium(III) triflate complexes, where one water molecule was detected in the inner coordination sphere, when the complex was dissolved in AN, in the case of samarium(III) triflate (and nitrate) complexes, no water molecule should be present in close proximity of the cation because the lifetimes observed in this solvent are relatively high (at least higher than those in the case of a MeOH solution). This conclusion is supported by the D_2_O/H_2_O exchange experiments in AN, described above for europium(III) complexes. Both for [Sm**L**(tta)_2_(H_2_O)]·CF_3_SO_3_ and [Sm**L**(tta)_2_]·NO_3_, the value of the samarium(III) lifetime does not change significantly upon the addition of 1 drop of D_2_O to the AN solution of the complexes [28.8(1) and 27.5(1) ms, respectively]. Nitrate and triflate complexes dissolved in the same solvent showed very similar luminescence lifetimes. The lower lifetime values recorded in MeOH are compatible with the presence of high-energy vibrations (OH) close to the metal center, capable of activating a multiphonon relaxation process. Accordingly, when the triflate and nitrate complexes are dissolved in CD_3_OD, the value of the samarium(III) lifetime increases (Table 3) in line with a CD_3_OD → MeOH substitution in the inner coordination sphere. A quick survey in the literature on the CPL activity of samarium(III) reveals that, after the first discovery of CPL in polar protic solvents (water and alcohols, typically) from this ion in 1986 (*g*_lum_ = 0.002),^21^ several steps forward have been made. Concerning chiroptical emission from chiral complexes, high values of *g*_lum_ have been reported for a naphthalene-based ligand containing a 2,6-pyridinedicarboxylic moiety.^25^ In this example, a *g*_lum_ value around 0.5 is reported at 560 nm. Samarium(III) cryptates containing bipyridine fragments show *g*_lum_ around 0.13 and a good value of the luminescence emission quantum yield (0.26%) in deuterated MeOH.^20^ Finally, it is interesting to note that samarium(III) complexes containing the ethylenediamine backbone show similar *g*_lum_ values (in the 0.03–0.06 range, around 560 nm) regardless of the chromophoric groups.^24,26^ The chiroptical performance of our complexes containing the chiral cyclohexanediamine backbone (see the *g*_lum_ values in Table 3) is in line with those recorded for the aforementioned complexes containing similar diamine backbones.

### Computational Results

**Figure 7:**
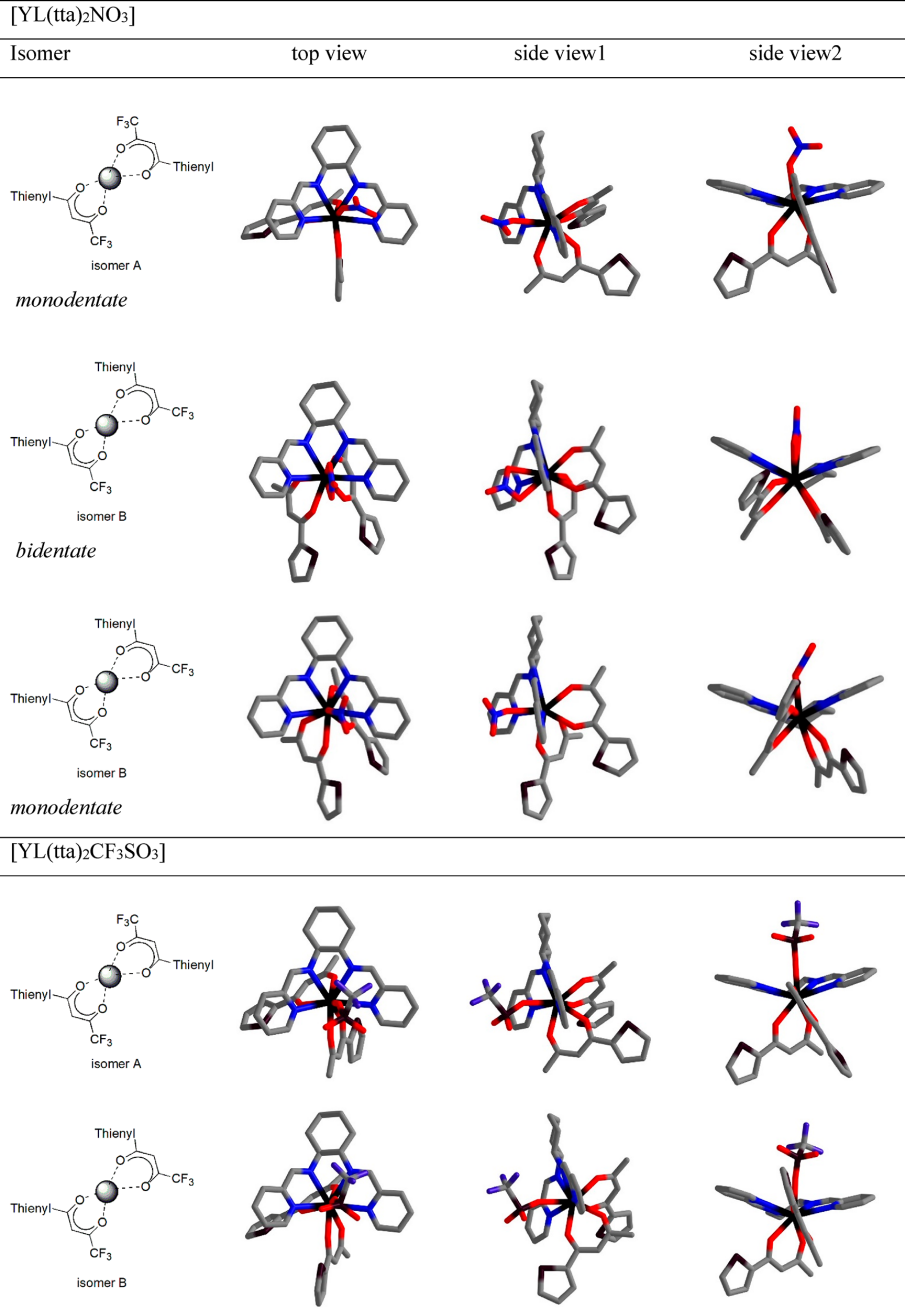
Minimum-energy structures of [Y**L**(tta)_2_A] complexes in DCM (H atoms omitted). Details about the structures of the A and B isomers are reported in Figure S1.

The ligand exchange Δ*G* values estimated according to the reaction (calculation for the nitrate complex was performed for isomer A with the nitrate ion coordinated to a monodentate mode)
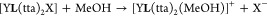
are −3.8 and −2.8 kcal mol^–1^ for nitrate and triflate, respectively. This result shows that replacement of the coordinated anions by MeOH is thermodynamically favored and a solvent-exchange equilibrium is likely to be present, as previously observed for the solvated ions and here suggested from the luminescence lifetime measurements. The minimum-energy structure of the [Y**L**(tta)_2_(MeOH)]^+^ complex is depicted in Figure S14. Even though disfavored in this solvent, at the complex concentration employed in the luminescence experiments, the presence of species containing the coordinated counterion should be taken into account, in particular in the case of the more coordinating nitrate. On the contrary, DCM species, in which the counterions are bound to the metal center, dominate the speciation. As shown in Figure 7, the minimum-energy structures in this solvent have been optimized, taking into account the usual coordination mode of nitrate (monodentate and bidentate) as well as the different relative orientations of the two tta ligands (isomers A and B, Figure 7).

In the case of the triflate complex ([Y**L**(tta)_2_]·CF_3_SO_3_), the CF_3_SO_3_^–^ anion is solely monodentate, thus with the metal ion 9-fold-coordinated (Figure 7). Because the Gibbs free energies for isomers C and D (Figure S1) are, in practice, the same as those of isomers B and A, respectively, from now on, we will discuss only the latter couple of isomers.

When the energies of the isomers are compared (Table 4), it is clearly evident that, in the case of a nitrate ion, the monodentate coordination mode is preferred. This applies to both solvents. Concerning the relative orientation of the two tta ligands, even though the A and B isomers possess similar energies in both solvents, we noticed a slight preference, which is stronger in MeOH for the nitrate complex, for the A isomer (Δ*G*_A,mono→B,mono_ = 1.1 and 0.6 kcal mol^–1^ in MeOH and DCM, respectively; Table 4). The same trend is observed when the [Y**L**(tta)_2_(MeOH)]^+^ complex in MeOH is investigated.

**Table 4:** Relative Stability (Δ*G*, kcal mol^–1^) of the Isomers Considered

	gas	DCM	MeOH
[Y**L**(tta)_2_NO_3_]			
A bi → B bi	–2.0		
B bi → A mono	–1.9	–3.2	–3.9
A mono → B mono	0.7	0.6	1.1
B bi → B mono	–1.2	–2.6	–2.8
[Y**L**(tta)_2_CF_3_SO_3_]			
A → B	0.5	0.7	0.8

This result indicates that different solvents not only cause changes in the degree of anion dissociation, as suggested by the experiments, but also are capable of influencing the A–B isomerization equilibria involving the tta ligands.

From a structural point of view, small differences in the bond distances between the A and B isomers are found in the case of [Y**L**(tta)_2_CF_3_SO_3_] (see Table 5 for the data in DCM).

**Table 5:** Relevant Bond Distances (Å) of the Minimum-Energy Structures of the [Y**L**(tta)_2_A] (A = NO_3_^–^ and CF_3_SO_3_^–^) Complexes in Figure 7 in DCM (MeOH in Parentheses) and for [Y**L**(tta)_2_(MeOH)] in MeOH^a^

	M–N_py_	M–N_im_	M–O_NO_3__	M–O_CF_3_SO_3__	M–O_MeOH_	M–O_tta_
[Y**L**(tta)_2_NO_3_]						
A mono	2.657 (2.650)	2.612 (2.604)	2.522 (2.563)			2.387 (2.367)
B bi	2.712 (2.712)	2.695 (2.689)	2.657 (2.677)			2.378 (2.379)
B mono	2.694 (2.698)	2.627 (2.623)	2.484 (2.513)			2.352 (2.352)
[Y**L**(tta)_2_CF_3_SO_3_]						
A	2.676 (2.678)	2.635 (2.631)		2.481 (2.502)		2.376 (2.356)
B	2.671 (2.673)	2.629 (2.626)		2.484 (2.505)		2.357 (2.358)
[Y**L**(tta)_2_(MeOH)]						
A	2.653	2.598			2.604	2.360
B	2.664	2.603			2.559	2.361

a Data for bonds of the same type are averaged.

These differences are more pronounced in the case of nitrate complexes; an elongation of the M–N bonds (0.01–0.08 Å, Table 5) is clearly observed when passing from A to B isomers in both solvents. Also, substitution of the counterions by the solvent molecule (MeOH) gives rise to a small change in the bond distances between the donor atoms and metal ion.

From the computational study, it is clear that multiple equilibria interconverting different species should take place. All of them display *C*_1_ symmetry, whereas NMR indicates effective *C*_2_ symmetry (equivalence of the two tta molecules and of the halves of the chiral ligand). This demonstrates that the NMR spectra are in all cases averages due to fast equilibria between the isomers, a situation that prevents any quantitative analysis of the paramagnetic shifts. Although there are structural differences between the various isomers, their relative energy display would be compatible with that of solution compositions, which combine in such a way to produce similar ECD spectra; i.e., they are not very different upon changes in the anion or solvent.

On the contrary, the nature of the further ligand (anion, solvent, and residual water), together with the different distances of the donor atoms listed in Table 5, justifies the argument, based on the crystal-field parameters, put forward above when discussing the difference in the CPL spectra. The complexity of the mixture in the case of the nitrate complex is even higher than that of triflate, particularly in the case of a MeOH solution, where at least six species are expected to be present (A bi, A mono, B bi, B mono, and the solvated A–MeOH and B–MeOH complexes). Further investigation on the anion binding as a function of the solvent and lanthanide is in progress.

## Conclusions

In this contribution, we demonstrate in detail the unexpected concurrent role of the counterion (triflate or nitrate) and solvent (DCM, AN, and MeOH) on the CPL activity of europium(III) and samarium(III) complexes containing tta and a tetraaza pyridine-based chiral ligand. This particularly applies to europium(III) complexes, where the CPL spectra of the species possessing the *same ligand stereochemistry* are nearly inverted when the employed solvents are DCM or MeOH. This effect could be connected with the presence of equilibria interconverting several isomers differing by the relative orientation of the tta ligands. As evidenced by DFT calculations, the difference between some bond lengths (M–N bonds, in particular) in the different isomers could be at the basis of such an unusual CPL activity. The results of the computational study also underline the high complexity of the solution, in particular in the case of MeOH where solvated and anion-coordinated complexes coexist. In the case of the europium(III) triflate complex, both the ^1^H NMR and CPL signals in AN retrace those calculated as a linear combination of the signals recorded in MeOH and DCM. This suggests that in AN significant amounts of the complex coexist with bound and dissociated triflate anions. To the best of our knowledge, this is the first case where achiral entities (counteranion and solvent) have such a strong effect on the CPL activity of chiral lanthanide(III) complexes, despite both their TL and ECD spectra being slightly affected.

## Supporting Information Available

Four possible relative orientations of the two tta ligands, UV–visible absorption, ECD, TL, CPL, room temperature decay curves, ^1^H NMR spectroscopic data, and minimum-energy structures (PDF)
