# Supplementary material for: Effect of the Counterion on Circularly Polarized Luminescence of Europium(III) and Samarium(III) Complexes
Source: Inorg Chem. 2020 Mar 18;59(7):5050–62. doi: 10.1021/acs.inorgchem.0c00280 (PMC7997384; doi:10.1021/acs.inorgchem.0c00280)
Supplement: Supplementary file 1 — ic0c00280_si_001.pdf [file ic0c00280_si_001.pdf]

Supporting Information

Effect of the counterion on Circularly Polarized Luminescence of Eu(III) and Sm(III) complexes

Lorenzo Arrico[1], Chiara De Rosa[2], Lorenzo Di Bari[1,*], Andrea Melchior[3], Fabio Piccinelli[2,*]

[1] Dipartimento di Chimica e Chimica Industriale, via Moruzzi 13, 56124 Pisa, Italy

[2] Luminescent Materials Laboratory, DB, Università di Verona, and INSTM, UdR Verona, Strada Le Grazie 15, 37134 Verona, Italy

[3] Dipartimento Politecnico di Ingegneria e Architettura, Laboratorio di Tecnologie Chimiche, Università di Udine, via Cotonificio 108, 33100 Udine, Italy

* corresponding author's email address: fabio.piccinelli@univr.it and lorenzo.dibari@unipi.it

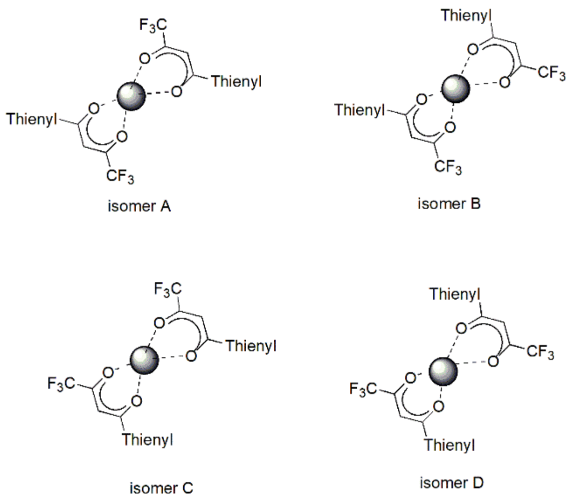

Figure S1. The four possible relative orientations of the two tta ligands. The ligand **L**, the counterion or the coordinating solvent are omitted for clarity. The metal center is symbolized by a gray sphere.

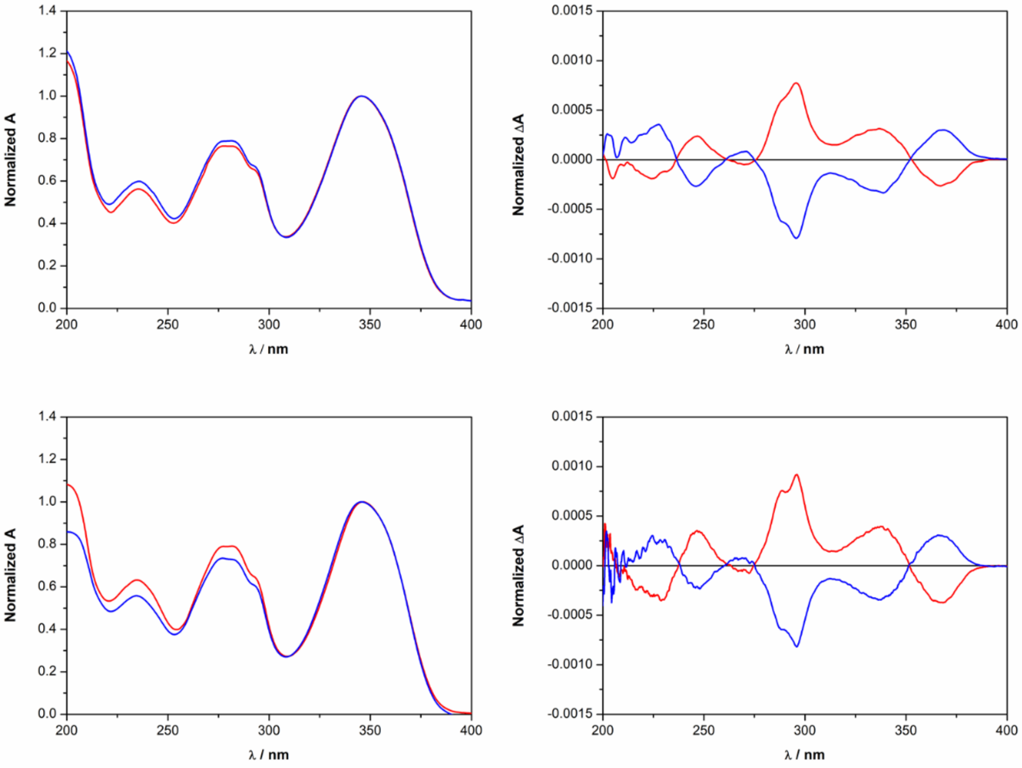

Figure S2. UV-visible Absorption (left) and ECD (right) spectra of [Eu**L**(tta)$_2$(H$_2$O)]·CF$_3$SO$_3$ (top) and [Sm**L**(tta)$_2$(H$_2$O)]·CF$_3$SO$_3$ in methanol.

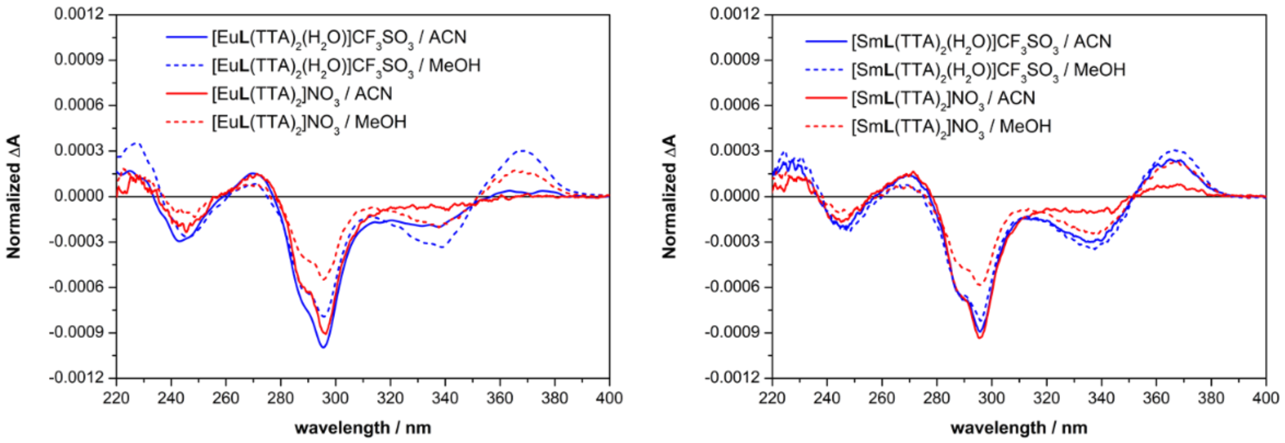

Figure S3. ECD spectra of the *R*,*R* Eu^{3+} (left) and Sm^{3+} complexes (right) with triflate (blue) and nitrate (red) dissolved in acetonitrile (continuous lines) and methanol (dashed lines).

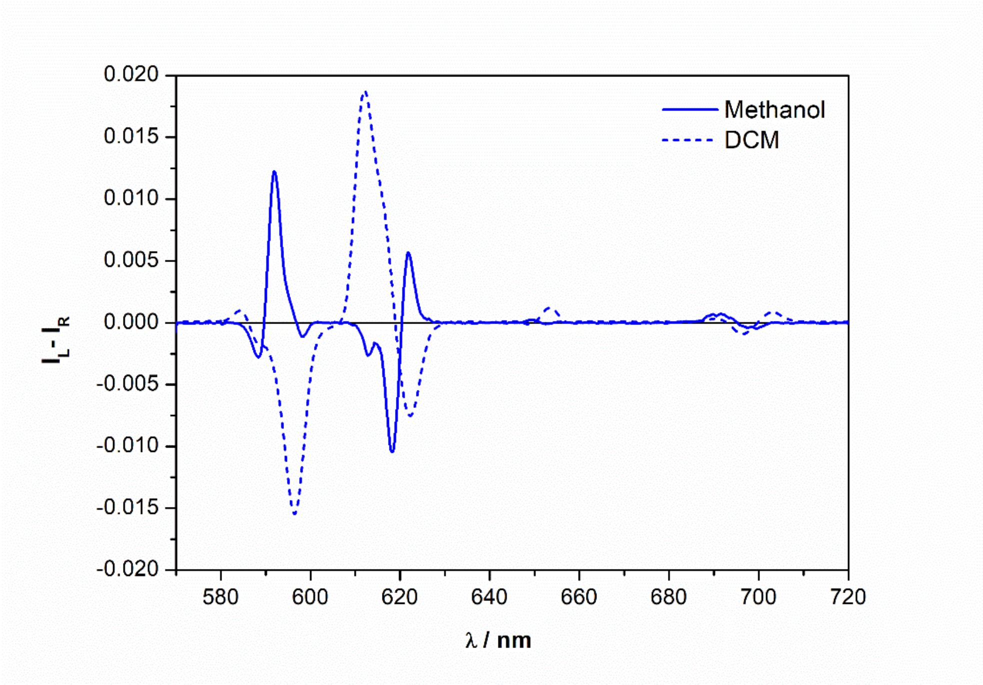

Figure S4. Comparison between the normalized CPL spectra of (*R*,*R*)-$[Eu\mathbf{L}(tta)_2(H_2O)]\cdot CF_3SO_3$ in methanol (blue line) and in dichloromethane (dashed blue line).

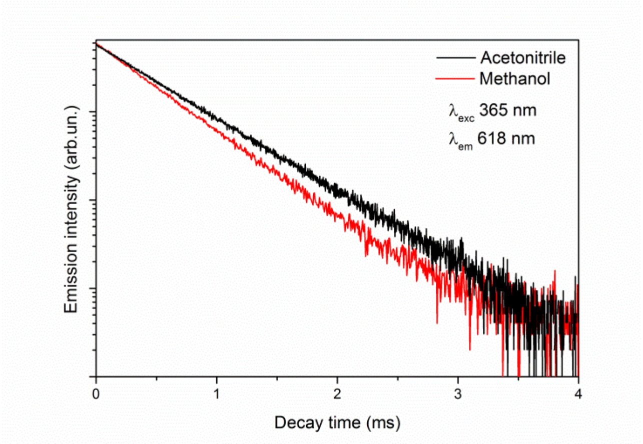

Figure S5. Room temperature decay curves of the $^5D_0$ Eu(III) emission excited around 365 nm in methanol and acetonitrile solutions for [Eu**L**(tta)$_2$]·NO_3. The decay curve of the complex in dichloromethane is not shown, as it is superimposable to that recorded in acetonitrile.

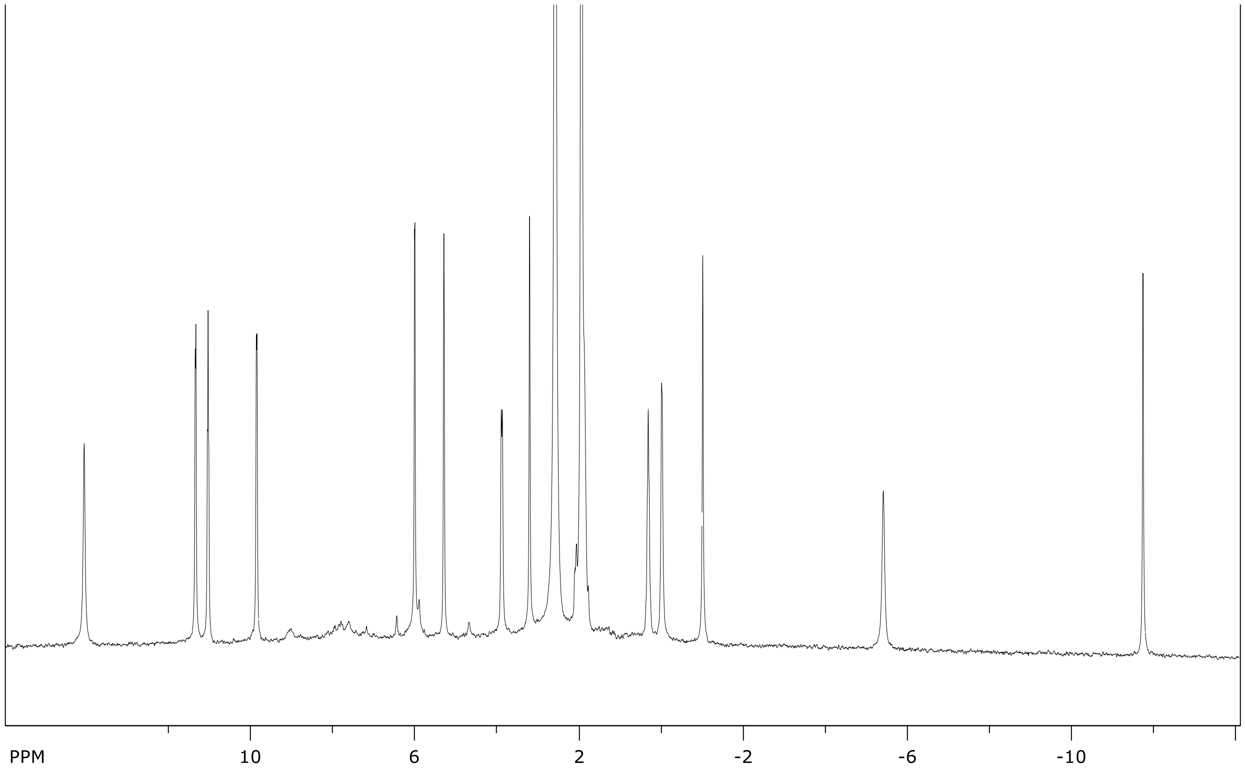

Figure S6. ^{1}H NMR spectrum of [Eu**L**(tta)$_2$(H_2O)]·CF_3SO_3 in acetonitrile-d$_3$.

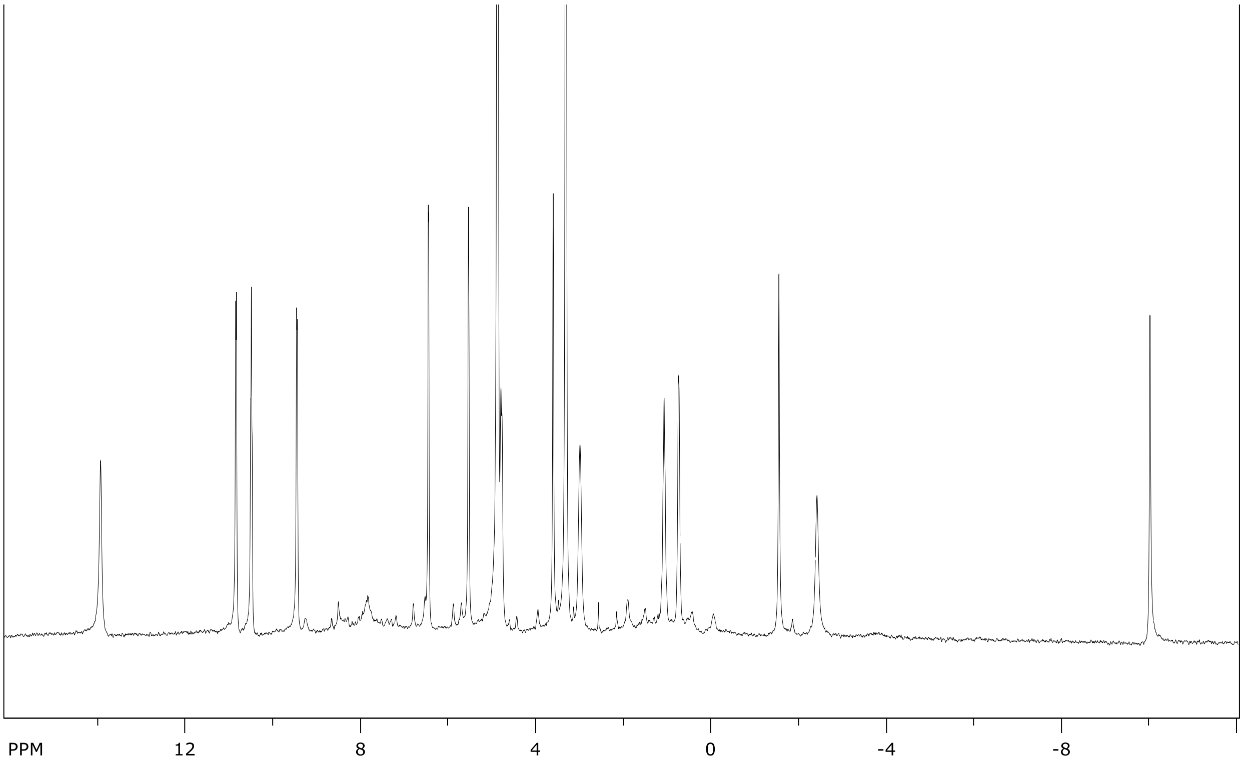

Figure S7. ^{1}H NMR spectrum of [Eu**L**(tta)$_2$(H$_2$O)]·CF$_3$SO$_3$ in methanol-d$_4$.

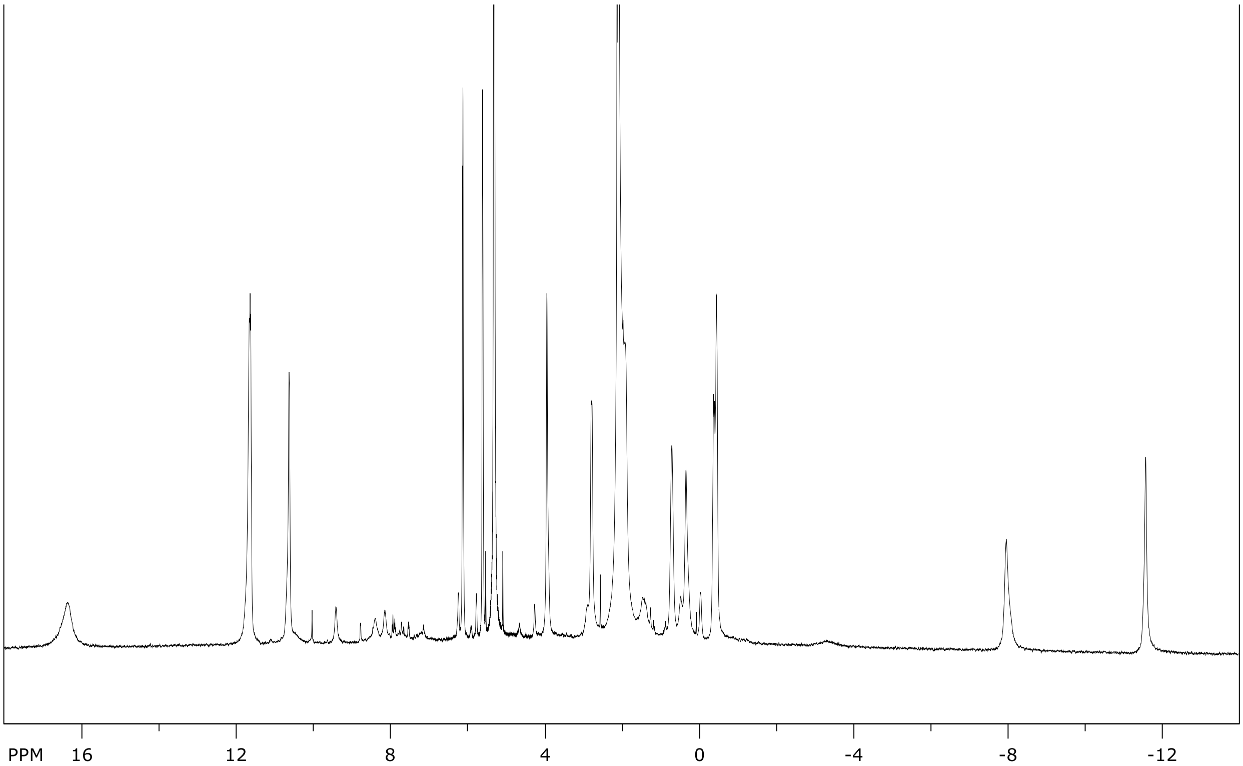

Figure S8. ^{1}H NMR spectrum of [Eu**L**(tta)$_2$(H$_2$O)]·CF$_3$SO$_3$ in dichloromethane-d$_4$.

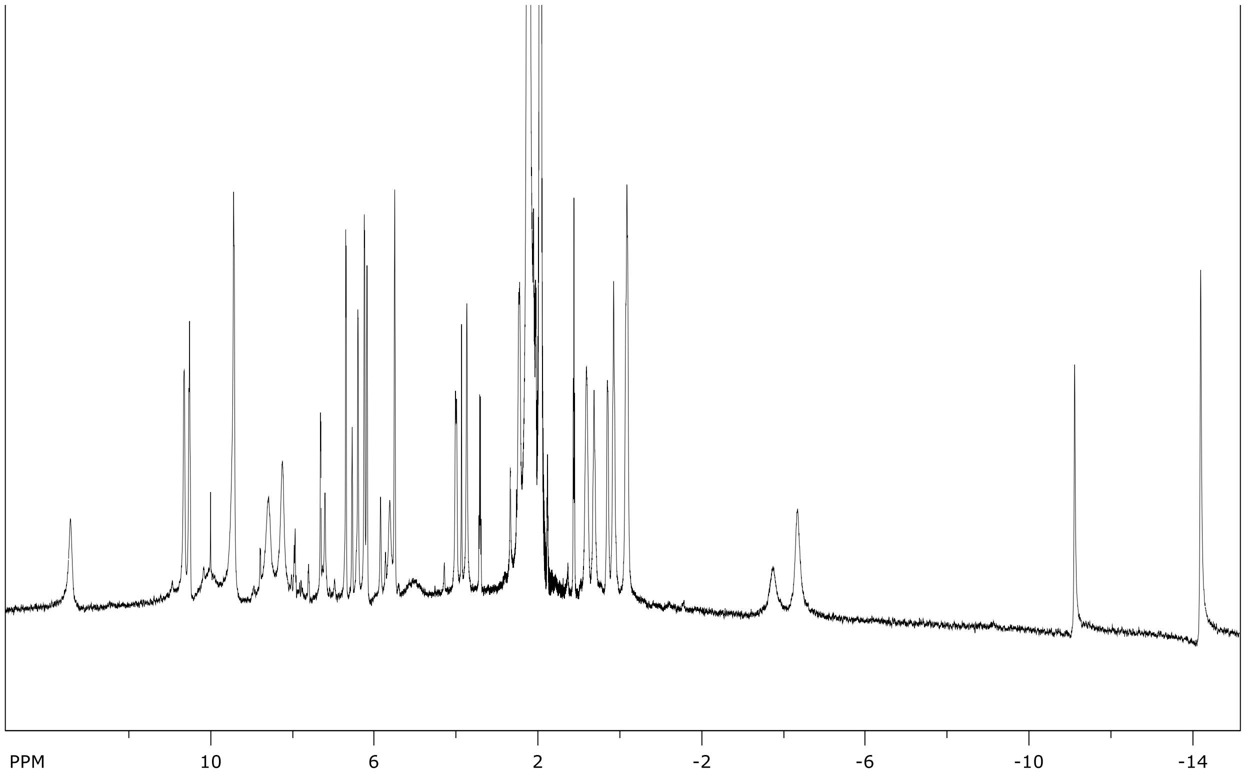

Figure S9. ^{1}H NMR spectrum of $[EuL(tta)_2]\cdot NO_3$ in acetonitrile-d_3.

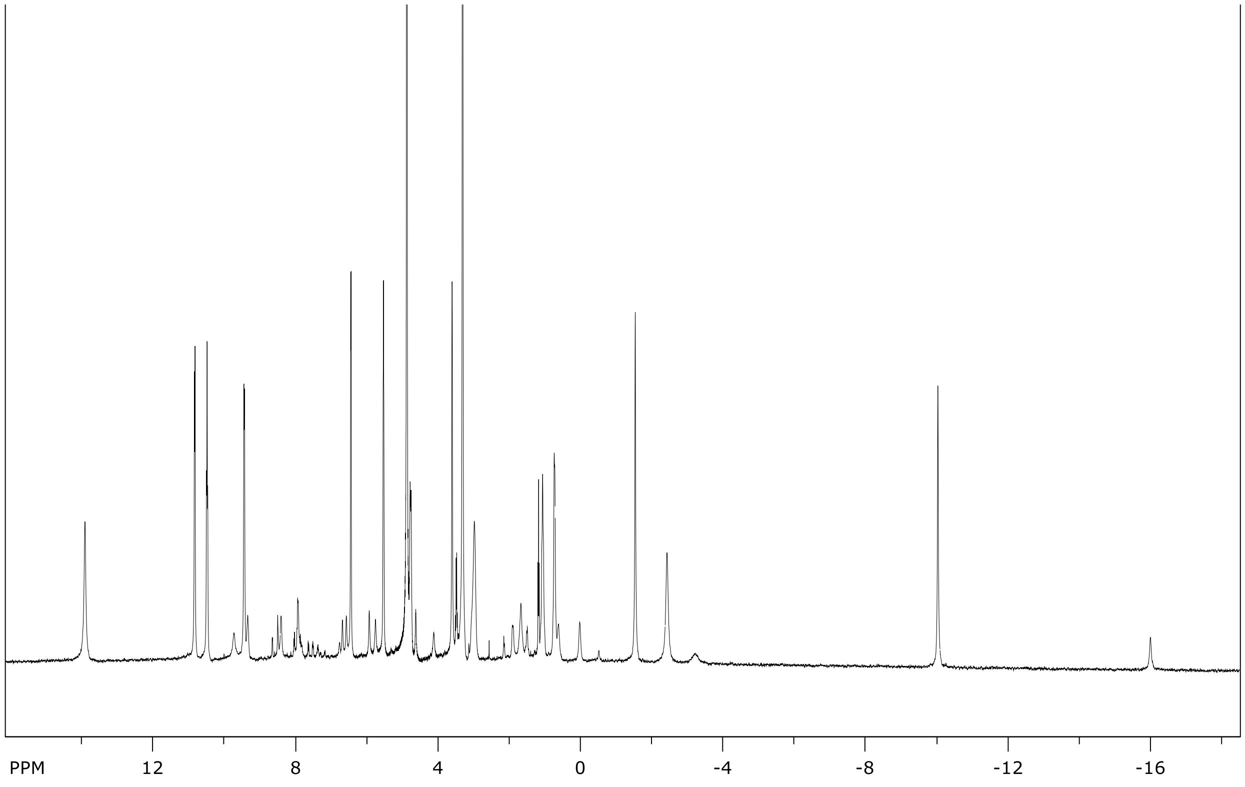

Figure S10. ^{1}H NMR spectrum of $[EuL(tta)_2]\cdot NO_3$ in methanol-d_4.

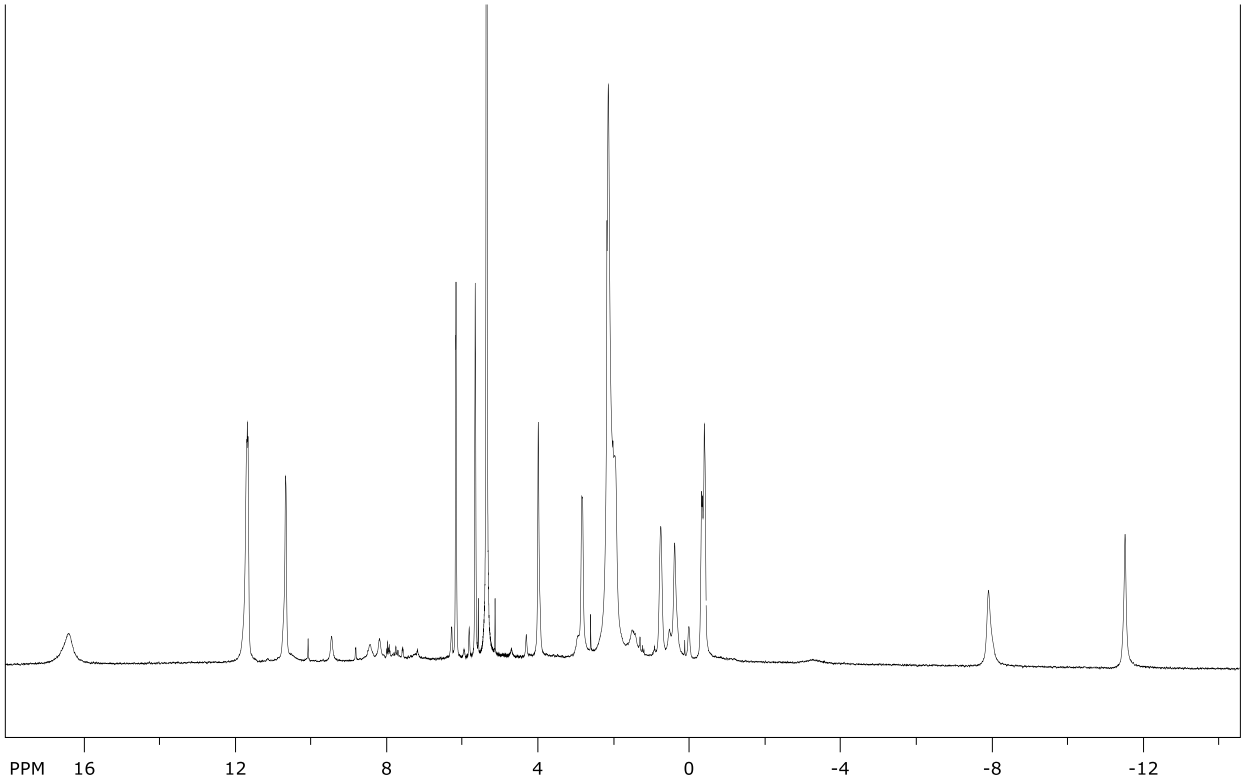

Figure S11. ^{1}H NMR spectrum of [Eu**L**(tta)$_2$]·NO_3 in dichloromethane-d$_2$.

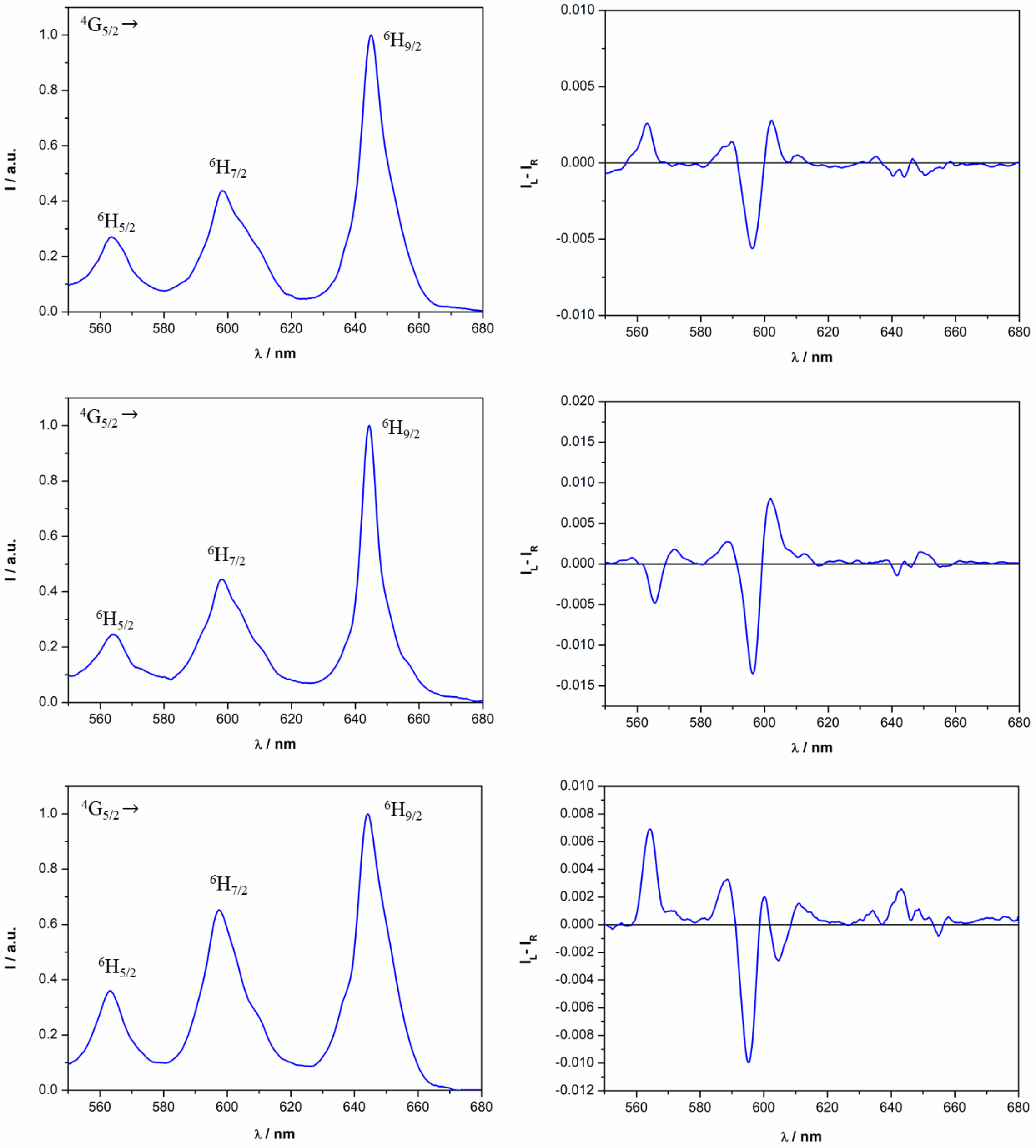

Figure S12. TL (right) and CPL(left) spectra of [Sm**L**(tta)$_2$]·NO_3 complex dissolved in acetonitrile (top), methanol (middle) and dichloromethane (bottom). The complexes with *R*,*R* stereochemistry are chosen as representative.

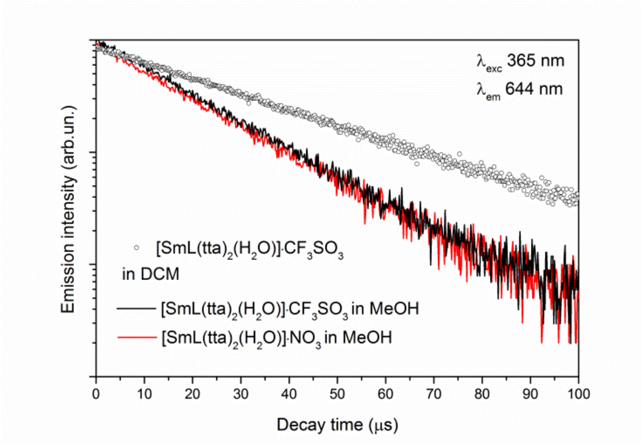

Figure S13. Room temperature decay curves of the $^4G_{5/2}$ Sm(III) emission excited around 365 nm in dichloromethane (DCM) and methanol (MeOH) solutions for [Sm**L**(tta)$_2$]·NO_3 and [Sm**L**(tta)$_2$(H_2O)]·CF_3SO_3.

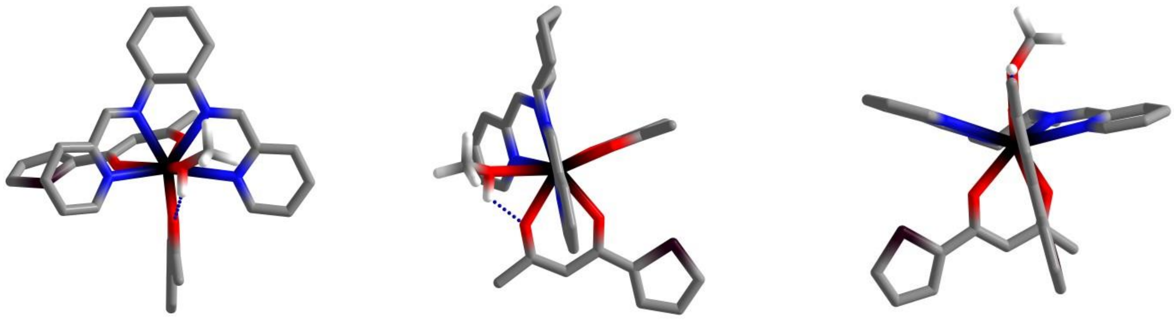

Figure S14. Minimum energy structures of [YL(tta)$_2$$CH_3OH$]$^+$ complex (H atoms omitted, left to right, top and side views).

Table S1. Experimental ^{1}H NMR chemical shifts (δ_{obs}) recorded in methanol-d$_4$ (δ_{obs}^{MeOD}), in dichloromethane-d$_2$ (δ_{obs}^{DCM}) and in acetonitrile-d$_3$ (δ_{obs}^{AN}) compared with the calculated[a] chemical shifts in acetonitrile (δ_{cal}^{AN}). For each chemical shifts, the difference between the experimental and calculated chemical shifts ($\Delta\delta = \delta_{obs}^{AN} - \delta_{cal}^{AN}$) is also reported.

δ_{obs} (ppm)			δ_{cal}^{AN} (ppm)	$\Delta\delta$
MeOD	DCM	AN		

13.93	16.37	14.07	14.71	-0.64
10.84	11.65[b]	11.35	11.10	0.25
10.49	11.65[b]	11.05	10.86	0.19
9.45	10.64	9.86	9.83	0.03
6.44	6.13	6.00	6.34	-0.34
5.53	5.62	5.29	5.56	-0.27
4.78	3.95	3.88	4.51	-0.63
3.6	2.80	3.20	3.34	-0.14
2.99	0.71	n.d.[c]	2.26	-
1.06	0.34	0.31	0.83	-0.52
0.73	-0.41[b]	-0.02	0.36	-0.38
-1.56	-0.41[b]	-1.02	-1.19	0.17
-2.43	-7.96	-5.42	-4.20	-1.22
-10.03	-11.57	-11.73	-10.52	-1.21

[a] $\delta_{cal}^{AN} = \chi^{MeOD}\delta_{obs}^{MeOD} + \chi^{DCM}\delta_{obs}^{DCM}$. The molar fractions χ^{MeOD} and χ^{DCM} were derived from the coefficients of the linear combinations obtained from the CPL spectra analysis. In particular, $\chi^{MeOD} = 0.38/(0.38 + 0.18) = 0.68$ and $\chi^{DCM} = 0.18/(0.38 + 0.18) = 0.32$.

[b] The integrals of the signals at 11.65 and -0.41 integrate twice respect to the others, thus they were split in two in order to perform the analysis of the chemical shifts.

[c] In the ^{1}H NMR spectrum in acetonitrile-d_3 only 13 signals (out of 14) could be clearly observed. Probably, the missing signal is overlapped with that of the residual solvent (1.94 ppm).
